# Expression quantitative trait loci analysis in rheumatoid arthritis identifies tissue specific variants associated with severity and outcome

**DOI:** 10.1136/ard-2023-224540

**Published:** 2023-11-18

**Authors:** Katriona Goldmann, Athina Spiliopoulou, Andrii Iakovliev, Darren Plant, Nisha Nair, Cankut Cubuk, The MATURA Consortium, Paul McKeigue, Michael R Barnes, Anne Barton, Costantino Pitzalis, Myles J Lewis

**Affiliations:** 1 Centre for Experimental Medicine & Rheumatology, William Harvey Research Institute, Queen Mary University of London, London, UK; 2 Centre for Population Health Sciences, Usher Institute, University of Edinburgh, Edinburgh, UK; 3 Centre for Genetics and Genomics Versus Arthritis, University of Manchester Centre for Musculoskeletal Research, Manchester, UK; 4 Centre for Translational Bioinformatics, William Harvey Research Institute, Queen Mary University of London, London, UK

**Keywords:** Arthritis, Rheumatoid, Polymorphism, Genetic, Synovitis, Pharmacogenetics, Methotrexate

## Abstract

**Objective:**

Genome-wide association studies have successfully identified more than 100 loci associated with susceptibility to rheumatoid arthritis (RA). However, our understanding of the functional effects of genetic variants in causing RA and their effects on disease severity and response to treatment remains limited.

**Methods:**

In this study, we conducted expression quantitative trait locus (eQTL) analysis to dissect the link between genetic variants and gene expression comparing the disease tissue against blood using RNA-Sequencing of synovial biopsies (n=85) and blood samples (n=51) from treatment-naïve patients with RA from the Pathobiology of Early Arthritis Cohort.

**Results:**

This identified 898 eQTL genes in synovium and genes loci in blood, with 232 genes in common to both synovium and blood, although notably many eQTL were tissue specific. Examining the HLA region, we uncovered a specific eQTL at *HLA-DPB2* with the critical triad of single-nucleotide polymorphisms (SNPs) rs3128921 driving synovial *HLA-DPB2* expression, and both rs3128921 and *HLA-DPB2* gene expression correlating with clinical severity and increasing probability of the lympho-myeloid pathotype.

**Conclusions:**

This analysis highlights the need to explore functional consequences of genetic associations in disease tissue. *HLA-DPB2* SNP rs3128921 could potentially be used to stratify patients to more aggressive treatment immediately at diagnosis.

**WHAT IS ALREADY KNOWN ON THIS TOPIC**

- Previous genome-wide association studies have identified numerous genetic loci associated with rheumatoid arthritis (RA) susceptibility.
- The strongest genetic association with RA is found in the human leukocyte antigen (HLA) region. Specific HLA alleles, particularly those in the *HLA-DRB1* locus, are strongly linked to an increased risk of developing RA.
- In RA, expression quantitative trait locus (eQTL) studies aim to understand how genetic variants influence the expression of specific genes in disease-relevant tissues, such as synovial tissue or blood.
- However, the functional effects of these genetic variants, their impact on disease severity and treatment response are still not fully understood.

**WHAT THIS STUDY ADDS**

- This study used eQTL analysis to investigate the relationship between genetic variants and gene expression in synovial tissue and blood samples from treatment-naïve patients with RA.
- 898 eQTL loci were identified in synovium, and 1251 eQTL were identified in blood, with 232 genes common to both tissues.
- Tissue-specific eQTLs underscore the importance of exploring genetic associations' functional consequences in disease tissue.
- Notably, a specific eQTL at *HLA-DPB2* was discovered, with single-nucleotide polymorphisms (SNP) rs3128921 driving synovial *HLA-DPB2* expression. Both rs3128921 and *HLA-DPB2* expression correlated with disease severity and lympho-myeloid pathotype, indicating a potential for immediate aggressive treatment stratification.

**HOW THIS STUDY MIGHT AFFECT RESEARCH, PRACTICE OR POLICY**

- The study advances our understanding of the functional implications of genetic variants in RA by pinpointing tissue-specific eQTLs.
- The identification of the *HLA-DPB2* SNP rs3128921 as a potential marker for disease severity and treatment response suggests a promising avenue for personalised treatment strategies.
- Clinically, the findings propose the use of *HLA-DPB2* SNP rs3128921 to guide aggressive treatment decisions early in the diagnostic process, potentially improving patient outcomes.

## Introduction

Rheumatoid arthritis (RA) is a systemic autoimmune disease characterised by joint inflammation, pain and damage, affecting 0.5%–1% of the population worldwide.^1^ Although both genetic and environmental factors can lead to RA, genetic components are known to be major determinants of susceptibility to the disease.^2^ One of the major challenges in human genetics is devising a systematic strategy to integrate disease-associated variants with diverse genomic and biological data sets to provide insight into disease pathogenesis and guide drug discovery. Genome-Wide Association Studies (GWASs) have uncovered many single-nucleotide polymorphisms (SNPs) associated with human traits. To prioritise causal genes, the associations between genetic variants and downstream molecular quantitative traits can be explored, the most proximal of which is gene expression. Colocalisation of an expression quantitative trait locus (eQTL) with a disease risk variant, therefore, implicates the gene as a candidate for disease causation.^3 4^

eQTLs (expression quantitative trait loci) vary by cell type and conditions of cell stimulation: only 30% of *cis*-eQTLs (loci proximal to the underlying gene) are consistently identified in different cell types from healthy donors.^5–8^ Therefore, eQTLs present in a specific cell type may not be detectable in another cell type or whole blood—and vice versa.^9^ Moreover, several eQTLs can be detected only under specific conditions of cell stimulation.^7^ This suggests that the contribution of eQTL data to inferred causality among candidate genes for a given disease must be understood at a cellular level and within a relevant biological context. An additional challenge in unpicking the relationship between genetics and disease is that disease susceptibility variants may differ radically from those which affect disease severity and outcome, not to mention drug response.^10^

In this study, we explore eQTL associations in synovial tissue and blood within treatment-naïve patients with RA as part of the Pathobiology of Early Arthritis Cohort (PEAC). There are several novel aspects to this study: first we look at eQTL in the disease tissue itself, using synovial biopsies and directly compare eQTL across disease tissue and blood; and second, we look for evidence of eQTL effects which link to clinical associations.

## Results

### *cis*-eQTL found for blood and synovium in early RA

**Figure 1:**
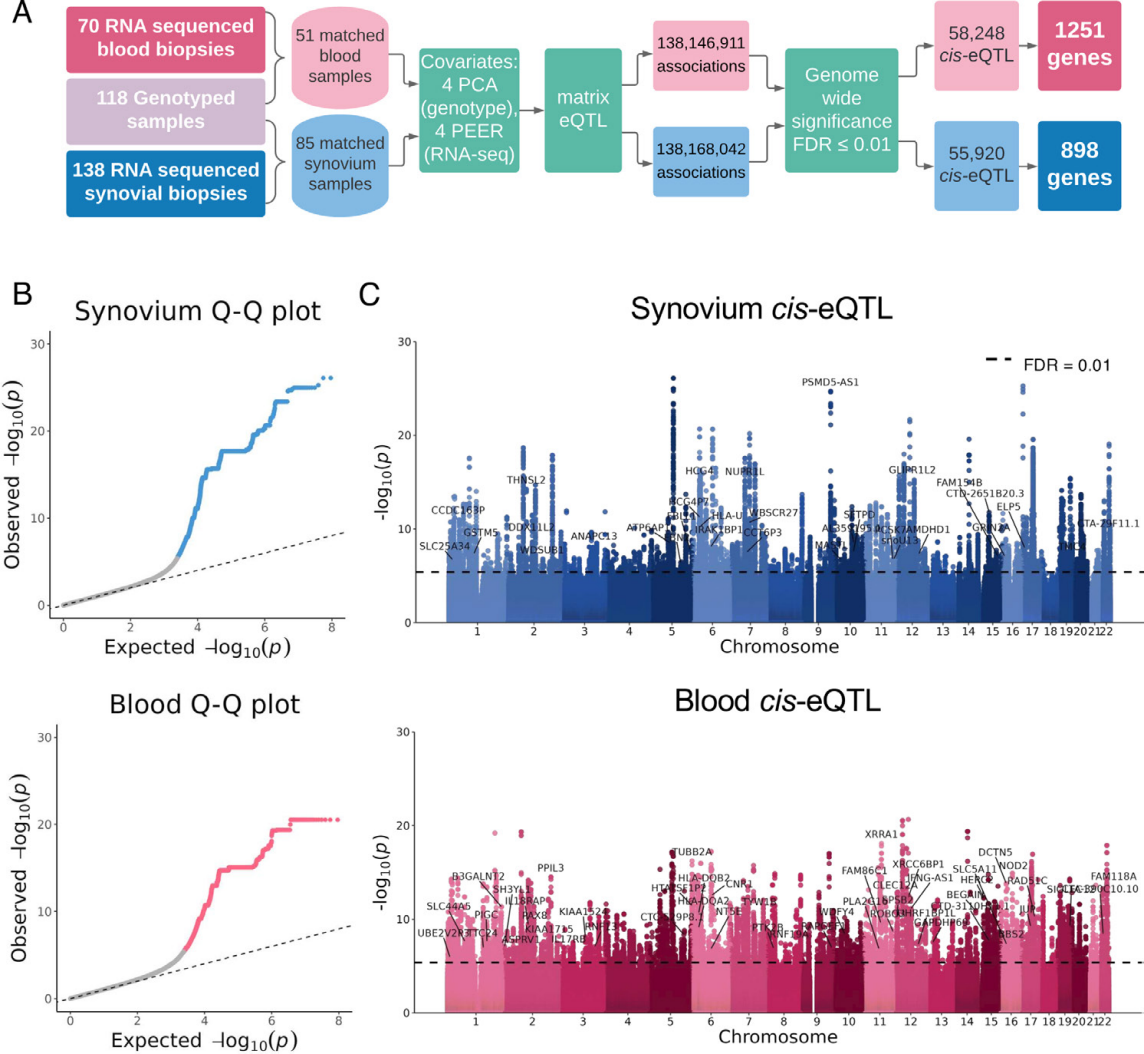
*cis-*eQTL for Blood and Synovium in Early Rheumatoid Arthritis *cis*-eQTL analysis was performed on 85 synovial and 51 blood samples using matrixEQTL (A). P values were calculated based on the t-statistic from a linear model with four PCA eigenvalues and four PEER factors as covariates. Following filtering of SNPs based on minor allele frequency (maf≥0.05), and genome wide significance (FDR≤0.01), 898 unique genes were found to have associated eQTL in synovium, and 1251 in blood. Log quantile-quantile (QQ) p value plots (B) indicate the overall level of significance from cis-eQTLs in each tissue. Manhattan plots (C) show genome-wide *cis-*interactions for variants located within ±0.5 Mb of genes in synovium (top) and blood (bottom). The dashed line represents genome-wide significance at FDR=0.01. Genes were labelled where there were over 50 significant gene-SNP interactions. eQTL, expression quantitative trait locus; FDR, false discovery rate; PCA, principal component analysis; PEER, Probabilistic Estimation of Expression Residuals; SNP, single-nucleotide polymorphism.

Matched genotype and RNA-seq data were available for 85 synovial samples, and 51 blood samples (characteristics and treatment are provided in online supplemental table 1). Using four genomic principal component analysis (PCA) eigenvectors (online supplemental figure 1) to adjust for ancestry, and four transcriptomic Probabilistic Estimation of Expression Residuals (PEER) factors as covariates to adjust for RNA-Seq batch effects, MatrixEQTL was used to identify cis-eQTLs based on p values calculated from linear models (figure 1A). This resulted in 55 920 significant synovial eQTLs and 58 248 in blood with a false discovery rate (FDR) of 1%. For synovial tissue this corresponded to 898 unique genes with significant eQTL signals, and 1251 in blood (online supplemental tables 2 and 3, respectively). Corresponding log quantile-quantile p value (Q-Q) plots for the eQTL analysis in each tissue (figure 1B) show the distribution of observed vs expected p values showing a lack of genomic inflation (genomic inflation factor λ_gc_=1.02 in synovium analysis and λ_gc_=1.04 in blood). *cis*-eQTL Manhattan plots (figure 1C) highlight the most significant genes in synovium (top) and blood (bottom) where the dashed lines represent genome-wide significance (FDR≤0.01).

10.1136/ard-2023-224540.supp2 Supplementary data

10.1136/ard-2023-224540.supp1 Supplementary data

Of the significant eQTL SNPs (eSNPs) and genes in both blood and synovial tissue, several overlapped with known GWAS-identified susceptibility associations for RA, autoimmune diseases or osteoarthritis (OA) according to the NHGRI-EBI GWAS catalogue^11^ (table 1). Locus plots for these eQTL genes are shown in online supplemental figures 2 and 3). Notably, several HLA eQTL genes were simultaneously identified in both synovial tissue and blood.

**Table 1:** Summary of genome-wide significant (FDR≤0.01) cis-eQTL genes in synovium and blood which have known autoimmune association for corresponding eSNPs in the GWAS catalogue

Leading eSNP	Chromosome	Eqtl gene	Beta	P value	FDR	GWAS associations of other eSNPS in locus
Synovium						
rs4690229:970724:A:T	4	DGKQ	0.23	4.07E-8	1.75E-4	AID+RA
rs251851:96325018:T:A	5	ERAP2	1.54	8.12E-27	3.74E-19	AID
rs11746807:150300419:G:A	5	ZNF300P1	1.13	1.97E-14	2.48E-10	AID+RA
rs416568:29647628:T:A	6	ZFP57	3.59	2.14E-21	2.34E-15	AID+RA
6:29897017:C:A	6	HLA-U	2.08	6.19E-11	4.90E-7	AID
rs1136903:29912280:T:C	6	HLA-J	−0.76	1.17E-11	1.00E-7	AID
rs73410515:29955610:G:A	6	HLA-V	3.22	9.66E-16	1.71E-11	AID
rs9270534:32559711:A:G	6	HLA-DRB9	−2.32	1.05E-16	4.41E-12	AID+RA
rs3104374:32600288:C:G	6	HLA-DQA2	1.72	1.11E-13	1.34E-9	AID+RA
rs35139945:32626267:G:T	6	HLA-DRB6	−2.81	3.63E-12	3.74E-8	AID+RA
rs9273497:32628297:T:C	6	HLA-DQB2	−1.35	3.18E-12	3.29E-8	AID+RA
rs6914616:33063592:C:A	6	HLA-DPB2	2.18	3.80E-13	4.39E-9	AID
rs465969:111655530:G:A	6	C6orf3	−1.02	2.66E-8	1.19E-4	RA
rs13290413:123517704:G:T	9	PSMD5-AS1	1.03	2.05E-25	1.05E-18	RA
rs4595501:115491565:T:A	10	CASP7	−0.35	1.30E-8	6.71E-5	AID
rs7943728:61547068:G:A	11	FADS2	0.86	4.94E-8	2.08E-4	RA
rs583887:65644027:T:C	11	CTSW	−0.88	5.59E-9	3.09E-5	RA
12:9874899:G:A	12	CLECL1	0.61	3.94E-6	9.77E-3	AID
12:56393337:C:G	12	RPS26	0.99	2.16E-22	3.79E-16	AID
rs140024366:28389173:G:A	16	CDC37P1	1.62	6.18E-9	3.38E-5	AID+RA
rs4072402:28937259:T:C	16	SULT1A2	0.60	2.46E-8	1.12E-4	AID
rs2693362:43657393:A:G	17	RP11-707O23.5	4.60	2.42E-19	9.47E-14	OA
rs112997627:43695263:T:G	17	DND1P1	2.15	8.11E-13	9.03E-9	OA
rs112836774:44084002:T:C	17	KANSL1-AS1	1.54	2.22E-19	9.27E-14	OA
rs112836774:44084002:T:C	17	CRHR1-IT1	0.88	2.02E-13	2.39E-9	OA
rs112836774:44084002:T:C	17	LRRC37A	0.92	1.19E-11	1.01E-7	OA
17:44215896:C:T	17	RP11-259G18.2	4.98	2.77E-20	1.25E-14	OA
17:44367588:TG:T	17	RP11-259G18.3	2.17	2.82E-18	1.45E-13	OA
Blood						
rs2235922:17416462:G:A	1	PADI2	−0.71	5.32E-15	1.42E-10	RA
rs7522061	1	FCRL3	0.43	2.46E-6	6.46E-3	RA
rs36068136:102989060:C:CT	2	IL18RAP	0.97	3.49E-10	2.90E-6	AID
rs1377586:961373:G:A	4	DGKQ	0.52	2.37E-12	3.53E-8	AID+RA
rs2161548:96318145:G:A	5	ERAP2	1.59	6.81E-18	4.38E-12	AID+RA
rs396660	6	ZFP57	2.82	2.15E-15	6.12E-11	RA
rs67692618:29701137:A:ATT	6	IFITM4P	1.08	2.95E-7	1.12E-3	AID
rs2743951	6	HLA-F	−0.33	5.42E-7	1.88E-3	AID
rs2734920:29883799:G:A	6	MICD	−1.58	3.23E-8	1.60E-4	AID
rs144398718:29904995:G:GA	6	HLA-J	1.00	2.87E-10	2.44E-6	AID
rs379221:29950140:A:G	6	HLA-V	3.31	1.06E-13	1.87E-9	AID
rs9271146:32577282:A:C	6	HLA-DRB9	−1.64	2.40E-9	1.51E-5	AID
rs9271385:32587409:A:G	6	HLA-DQA2	−1.76	7.15E-10	5.00E-6	AID+RA
rs9272346:32604372:G:A	6	HLA-DQB1	0.94	2.50E-10	2.14E-6	AID
rs707951:32609813:T:C	6	HLA-DQB2	−1.92	2.05E-11	2.14E-7	AID
rs9273007:32611537:C:T	6	HLA-DRB6	1.26	3.12E-10	2.63E-6	AID
rs6914616:33063592:C:A	6	HLA-DPB2	2.10	2.60E-11	2.68E-7	AID
rs6925764:34832661:T:C	6	UHRF1BP1	0.33	6.76E-10	4.75E-6	AID
rs33980500	6	C6orf3	−1.08	5.29E-8	2.49E-4	RA
rs35368136:167407596:CT:C	6	RNASET2	−0.53	1.86E-12	2.81E-8	AID
rs1382568:11351220:A:C	8	FAM167A	1.17	2.76E-9	1.70E-5	AID+RA
rs6478109	9	TNFSF15	0.90	9.27E-7	2.96E-3	AID+RA
rs2590311:59983565:A:G	10	CISD1	−0.46	1.68E-10	1.50E-6	AID
rs7943728:61547068:G:A	11	FADS2	2.33	3.02E-16	4.62E-11	RA
rs499425:64105929:G:A	11	AP003774.1	0.76	5.21E-7	1.82E-3	AID
12:56435929:C:G	12	RPS26	1.44	2.24E-21	1.08E-14	AID
rs8049439	16	TUFM	0.26	5.86E-14	1.37E-9	AID
rs34492203:68577766:CT:C	16	ZFP90	−0.28	1.10E-9	7.40E-6	AID
rs638416:18266975:G:C	17	SHMT1	−0.35	8.80E-9	4.98E-5	AID
rs8069176:38057197:G:A	17	ORMDL3	−0.34	9.38E-13	1.46E-8	AID+RA
17:43663455:C:CT	17	LRRC37A4P	−0.89	1.22E-7	5.16E-4	OA
17:43667635:A:G	17	DND1P1	2.85	4.88E-14	1.14E-9	OA
17:43947883:T:G	17	RP11-259G18.2	2.62	2.33E-12	3.48E-8	OA
17:43965129:AT:A	17	KANSL1-AS1	2.28	1.00E-12	1.56E-8	OA
rs17564493:44001379:C:T	17	RP11-707O23.5	3.36	6.97E-17	3.02E-11	OA
rs55649944:44119463:C:T	17	RP11-259G18.3	2.55	1.03E-16	4.01E-11	OA
rs67919208:45771873:T:C	17	TBKBP1	0.37	8.13E-7	2.64E-3	RA
rs1143438:46889161:G:A	19	PPP5C	−0.37	3.95E-12	5.72E-8	RA
rs4821544	22	PVALB	−1.81	2.12E-11	2.20E-7	AID

AID, autoimmune disease; FDR, false discovery rate; GWAS, Genome-Wide Association Studies; OA, osteoarthritis; RA, rheumatoid arthritis; SNP, single-nucleotide polymorphism.

In order to uncover functional effects of synovial and blood eQTL genes with biological relevance to RA pathogenesis, we examined enrichment of transcription factors and diseases among eQTL genes in each tissue using topGO with a Kolmogorov-Smirnov test (online supplemental figure 4A,B). Multiple autoimmune disease pathways were enriched in synovial tissue including antigen processing and presentation, MHC protein complexes and interleukin (IL) receptor binding. This suggests that synovial eQTL genes are more antigen and disease specific. Blood eQTL genes on the other hand were enriched for innate immune response and NF-kappaB pathways. This is consistent with blood related eQTL genes being associated more generally with amplification of acute inflammation.

### eQTL variants associate with response to disease-modifying antirheumatic drugs

**Figure 2:**
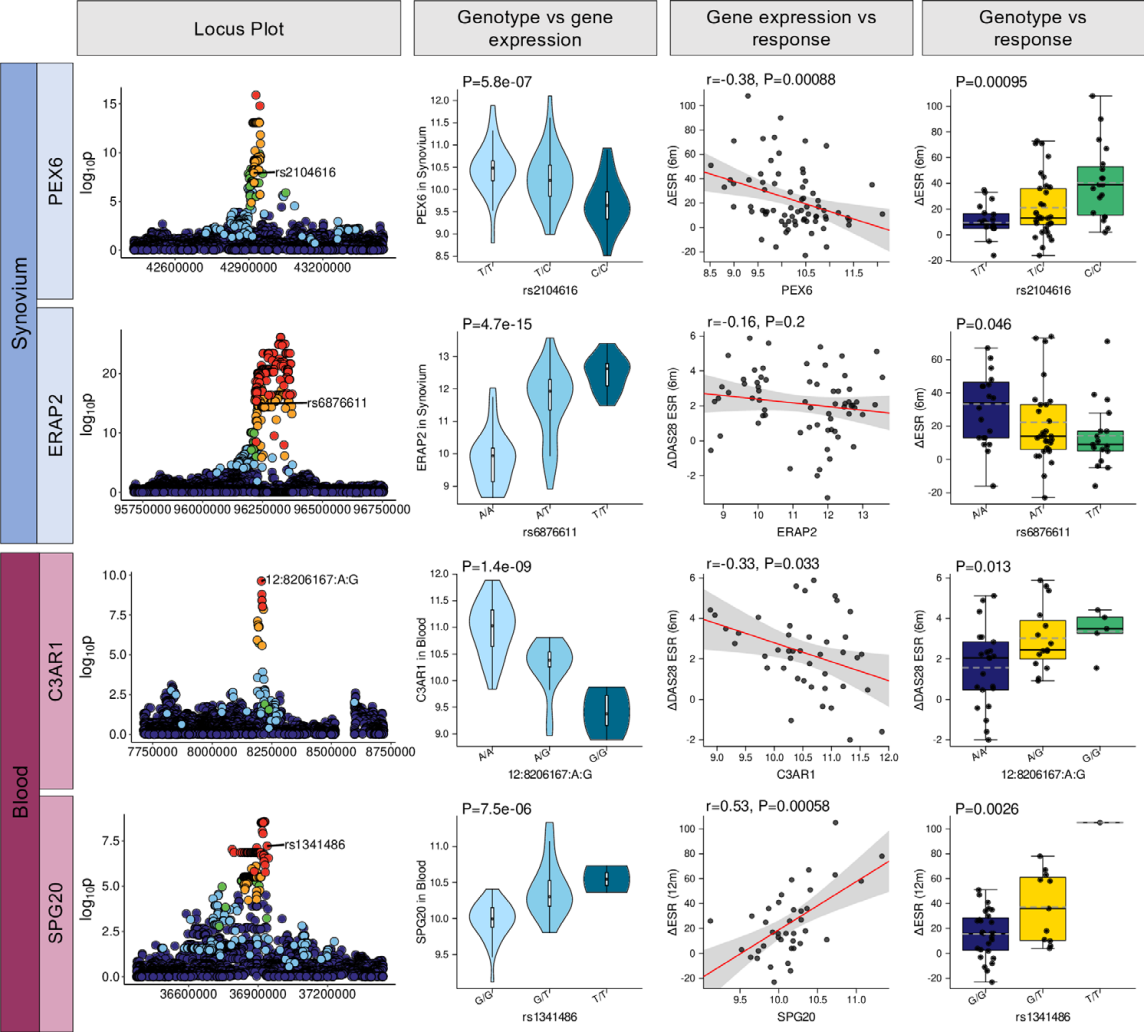
Selection of eQTL SNPs and genes associated with clinical response. Columns are ordered from left to right. The first column (far left) shows locus Manhattan plots for representative eQTLs with -log_10_ p value on the *y* axis and SNP chromosomal position on the x axis. P values were calculated based on the t-statistic from a linear model with four genotype eigenvalues and four RNA-Seq PEER factors as covariates. Synovial eQTL at the *PEX6* and *ERAP2* loci, and blood eQTL at the *C3AR1* and *SPG20* loci are shown. The second column shows violin plots of expression of the most strongly associated eQTL SNP and baseline RNA-Seq gene expression in either synovium or blood (statistical analysis by linear model with four PCA andfour PEER factor covariates). The third column shows relationships between baseline RNA-Seq gene expression and clinical outcome measures: change in ESR or DAS28-ESR between baseline and 6 months following methotrexate-based DMARD therapy (statistical analysis by Spearman’s correlation). The fourth (far right) column shows the relationship between the eQTL SNP and the clinical outcome measure: rs2104616, rs6876611 and rs1341486 are linked to change in ESR (right), whereas 12:8206167:A:G is associated with change in DAS28-ESR (statistical analysis by linear model with four genotype PCA covariates). For each of these SNPs, the corresponding eQTL gene (with the exception of *ERAP2*) is also associated with the response variable. DAS28, Disease Activity Score in 28 joints; DMARD, disease-modifying antirheumatic drug; ESR, erythrocyte sedimentation rate; eQTL, expression quantitative trait locu; PCA, principal component analysis; PEER, Probabilistic Estimation of Expression Residuals.

To explore downstream effects of eQTL in response to disease-modifying antirheumatic drug (DMARD) treatment, linear regression models were used to highlight significant associations between eSNPs and RA clinical response after 6 months of treatment. Clinical response was measured as change in erythrocyte sedimentation rate (ESR), change in C reactive protein (CRP), change in Disease Activity Score in 28 joints (DAS28) and EULAR response criteria comparing DAS28 and subcomponents at baseline and following 6 months of methotrexate-based combination DMARD therapy. Following multiple testing (FDR≤0.05), there were 15 eSNP-response associations in synovium and nine in blood (online supplemental table 4). Interestingly, in synovial tissue several *PEX6* eSNP correlated with change in DAS28_ESR_ and change in ESR (figure 2). This association was also observed at the gene expression level. Additionally, *ERAP2* SNPs were associated with the change in ESR, although not at the gene expression level. In blood, *C3AR1* eSNPs were inversely correlated with gene expression, which was also associated to change in DAS_ESR_. *SPG20* eSNP genotypes and gene expression in blood was associated with change in ESR.

### eQTL genes indicative of protein-QTL effects

To explore if the SNPs affecting synovial gene expression also affect protein level, we used the GENOSCORES platform (https://genoscores.cphs.mvm.ed.ac.uk)^10^ to compare genotypic scores for synovial gene expression (eQTL scores) with genotypic scores for circulating protein levels (pQTL scores), the latter computed using publicly available pQTL summary statistics for 1478 circulating proteins from the INTERVAL study.^12^ To determine if the same underlying haplotypes drive the signals for gene expression and protein levels, we computed genetic correlations between eQTL and pQTL scores located within 200 kb from each other. An eQTL score-pQTL score correlation above 0.5 within a locus window was used as suggestive evidence of a shared genetic signal (see also online supplemental methods).

Among the genes with a synovial cis-eQTL score, 111 were located within 200 kb of a pQTL score. From these, 27 gene–protein pairs had eQTL-pQTL score–score correlation that exceeded 0.5 (r>0.5) (online supplemental table 5). Pathway enrichment analysis of significant proteins using the UniprotR R package revealed notable regulation of histone pathways (Online supplemental figure 5), indicating potential genetic variants driving epigenetic modification. Additionally, upregulation of *IL27* and *IL35* mediated pathways, as well as regulation of haemopoiesis, was observed. However, it is important to note that this analysis has limitations due to the limited protein QTL data available (summary statistics on 1478 circulating proteins), and further in-depth quantitative proteomic studies are necessary for comprehensive coverage of the entire proteome.

### Synovial trans-eQTL suggestive of downstream effects related to immune regulation

**Figure 3:**
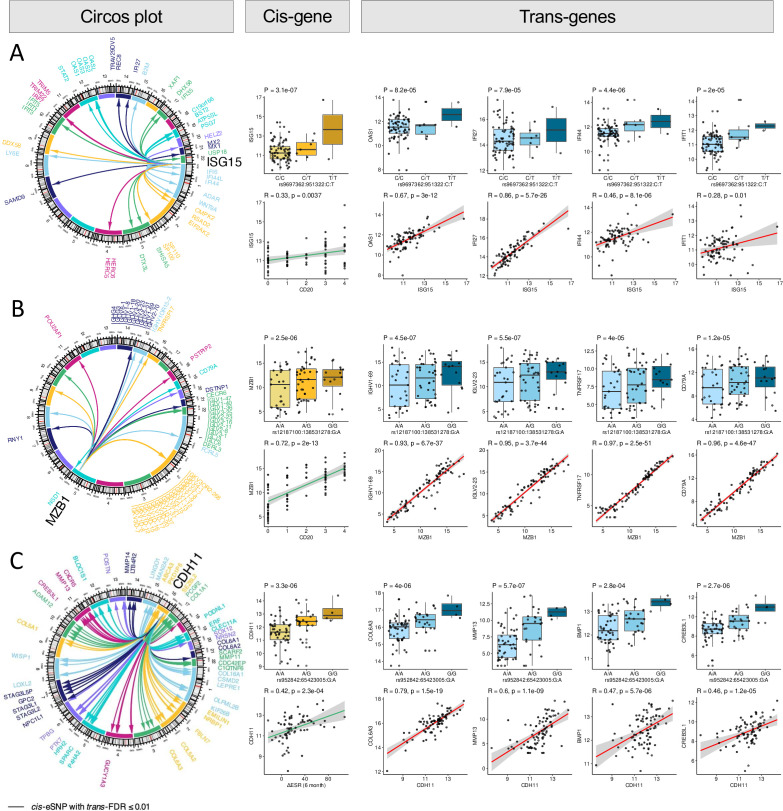
Selected trans-eQTL effects from leading *cis-*eSNPs The leading synovial cis-eSNPs were used in a synovial tissue trans-eQTL analysis. Several genes associated with RA disease shared common significant variants. Circos plots, left, show the links between the source SNP and its effect on different trans-eQTL genes across the genome. Boxplots indicate associations between genotype and gene expression (statistical analysis by linear model with four genotype PCA and 4 PEER factors as covariates using matrixEQTL). Scatter plots show correlation between the cis-eQTL gene and trans-eQTL gene (statistical analysis by Spearman’s correlation). (A) SNPs at the *ISG15* locus correlated both with baseline *ISG15* expression as well as downstream interferon stimulated genes *GAS1*, *IFI44* and *IFIT1* and synovial B cell infiltration measured by CD20 histology. (B) SNPs at *MZB1* (marginal zone B and B1 cell specific protein) correlated with synovial immunoglobulin gene synthesis and the B-cell related gene *CD79A*. (C) SNPs at the *CDH11* (cadherin 11) locus demonstrated a trans-eQTL correlating with collagen genes and matrix metalloprotease-13 (*MMP13*). *CDH11* trans-eQTL SNPs also correlated with clinical response as demonstrated by change in ESR at 6 months following methotrexate-based DMARD therapy. DMARD, disease-modifying antirheumatic drug; ESR, erythrocyte sedimentation rate; eQTL, expression quantitative trait locu; PCA, principal component analysis; PEER, Probabilistic Estimation of Expression Residuals.

Having performed a *cis-*eQTL analysis, we scrutinised the data from synovial tissue further by performing a linear regression *trans-*eQTL analysis for the leading *cis-*eSNP for each eQTL gene using matrixEQTL with four genotype PCA and four PEER factors as covariates. Trans-eQTL analysis presents challenges due to potential false positives from numerous permutations. However, we observed several trans-eQTL with multiple SNP hits from the same locus associated with a particular gene, thus increasing the likelihood of these trans-eQTL results being genuine. Genes of biological interest are highlighted in figure 3 and Online supplemental figure 6). Notably *ISG15* eSNPs were significantly associated with altering expression of well-known type 1 interferon response genes (*IFI27, IFI35, IFI44, IFIT1, IFIT3, IFIT5* and *OAS1*, figure 3A).^13^ The *ISG15* transEQTL also correlated with synovial B cell infiltration as measured by CD20 histology consistent with our previous finding that type 1 interferon gene expression signature was associated with synovial B cell infiltration.^14^ A similar blood interferon gene signature has been reported by Cooles *et al* as a prognostic biomarker in early RA individuals treated with methotrexate-based DMARDs.^15^

eSNPs at *MZB1* (marginal zone B and B1 cell specific protein), which is specifically expressed in B cells, correlated with the expression of other B-cell related genes, including immunoglobulins (IGH, IGK, IGL), *CD79A* (which has previously been associated with joint destruction in RA,^16^ *TNFRSF17*,^17^ *FCRL5* 18 and *POU2AF1* (figure 3B). Trans-eQTL effects were also observed for eSNPs at the *CDH11* (Cadherin 11) locus, which is associated with osteoblast differentiation^19 20^ and synovial lining fibroblast subtype.^21^ eSNPs which drove *CDH11* expression also correlated with multiple genes involved in bone development/metabolism (figure 3C): collagens (eg, *COL1A1*, *COL3A1*, *COL5A1* and *COL6A1*)^22^; matrix metallopeptidase genes (eg, *MMP11*, *MMP13* and *MMP14*)^23^; the cartilage-inducing gene *BMP1* (bone morphogenetic protein-1)^24^; *NOTCH3* which regulates sublining fibroblasts^25^; and *CREB3L1*. This suggests these variants around *CDH11* may play a role in modulating abnormal fibroblast development in synovial tissue. *CDH11* transEQTL SNPs also correlated with clinical response as demonstrated by change in ESR at 6 months following methotrexate-based DMARD therapy.

Additionally, eSNPs linked to the chemokine *CXCL9* were associated with T-cell specific trans-eQTL effects for *PPARG* (PPAR gamma) and *GZMA* (granzyme A) which is expressed in CD8 synovial T cell subsets^26^; kinases (*CDK2AP2*), and immunoglobulins (*NCAM2*) (online supplemental figure 6A). Similarly, eSNPs of *DAPK1* (death associated protein kinase 1) which mediates gamma-interferon induced cell death were highly associated with additional kinases (eg, *CDK1*); fibroblast genes (*FGFR1*); phosphatases (*DUSP10* and *PTPRS*); the erythropoietin receptor *EPOR* which activates JAK/STAT pathways^27^; and genes relating to TNF (eg, *CCDC3*,^28^ *TNFSF13*, *C1QTNF7* and *TNFAIP1*) (online supplemental figure 6B).

### Tissue differentiating eQTL between blood and synovial tissue

**Figure 4:**
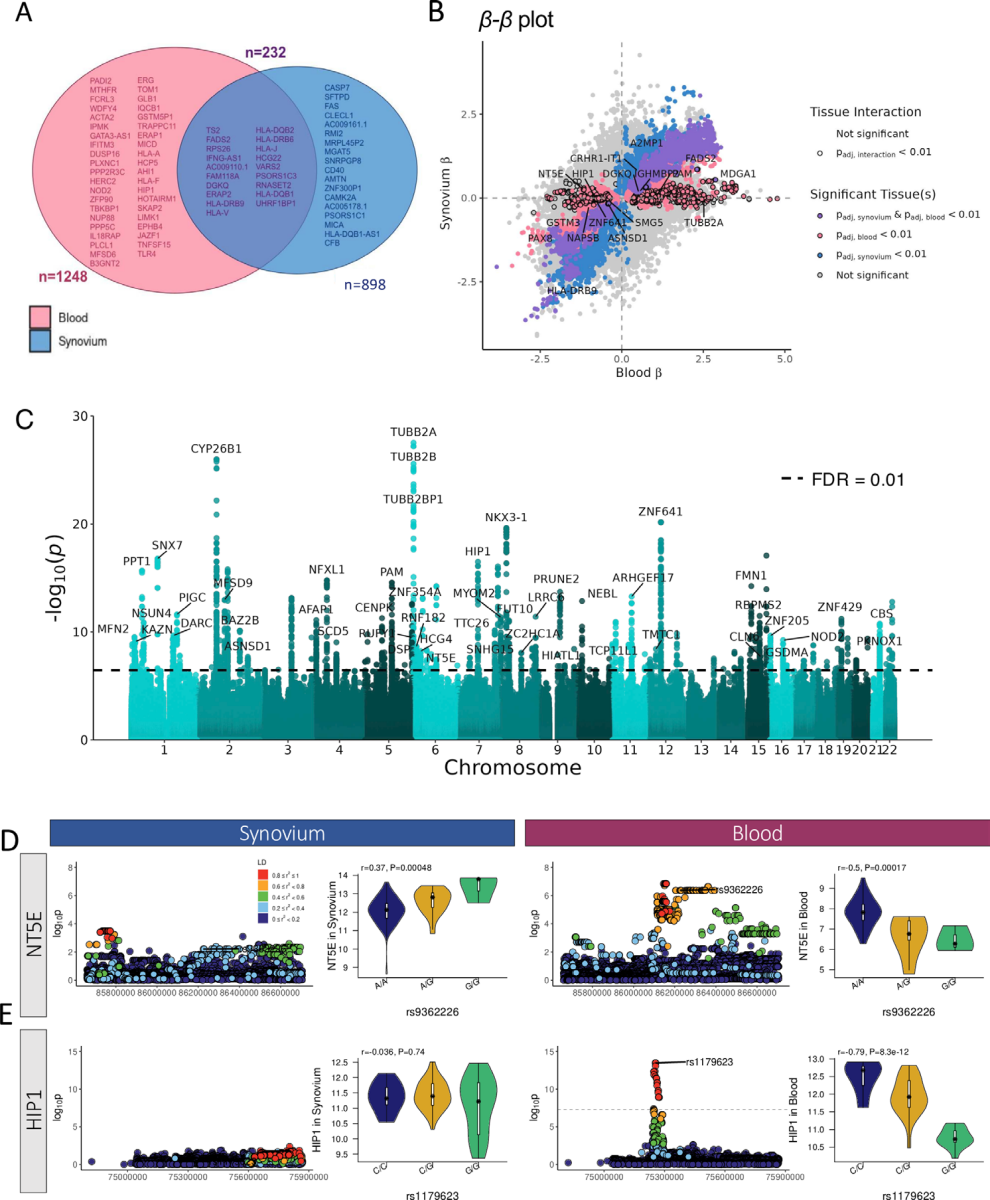
Differences and interactions between synovium and blood eQTL (A) Venn diagram of genome-wide significant genes in synovium and blood with those of known autoimmune disease association in the GWAS catalogue labelled. There are 232 genes which are common to both synovial and blood eQTL. (B) Beta-beta plots highlighting gene-SNP pairs whose eQTLs show an opposite effect in synovium compared with blood. Pairs are coloured according to significance in each tissue and outlined if tissue interaction is significant. (C) Manhattan plot showing the p values for the linear synovial-blood tissue interaction. Significant genes (FDR≤0.01) highlight differences in eQTL between tissues. (D–E) Examples of eQTL effects differing between tissues shown by eQTL locus plots and violin plots for expression in synovium and blood, p values calculated by linear model: *NT5E* significant tissue interaction; and *HIP1* significant in blood only. eQTL, expression quantitative trait locu; FDR, false discovery rate; SNP, single-nucleotide polymorphism.

A total of 232 eQTL genes were identified in both blood and synovial tissue, with known autoimmune traits labelled in figure 4A. The Jaccard similarity coefficient comparing the number of common significant genes between the two tissues was 0.12, indicating relatively low overlap. The multitissue (MT-eQTL) analysis (outlined in references 29 30) was performed to combine eQTL results in synovium and blood to detect differences between tissues. Additionally, to determine the statistical differences between tissues, the matrixEQTL pipeline was repeated using tissue as an interaction term. The beta-beta coefficient plot (figure 4B) highlights SNP-gene pairs with FDR≤0.1 in both tissues (purple) and those exhibiting significant tissue interaction or specificity (black). Figure 4C represents the significance of tissue interaction, with lower p values indicating larger differences in the eQTL effect between tissues for a given SNP–gene pair. The gene *NT5E*, involved in joint and arterial calcification,^31–33^ demonstrates opposing beta values in each tissue (figure 4D). It is a significant eQTL gene in blood, but not synovial tissue. The genotype for the most significant blood eSNP (rs9362226) highlighted the opposing direction of the effect in each tissue.

β-β plots revealed significant associations in at least one tissue, indicated by the top-left and bottom-right quadrants, revealing eQTLs which demonstrated an opposite effect in synovium compared with blood. This is critically important as it shows that SNP variants can drive completely opposing patterns of gene expression in tissue compared with peripheral blood. One gene which exemplifies this pattern: *HIP1* (huntingtin interacting protein 1), which is associated with systemic lupus erythematosus (SLE)^34^ and RA synovial fibroblasts^35 36^ (figure 4E).

### PEER covariate associated with several eQTL Genes

To investigate the effect of histological pathotype in synovium, previously reported by Lewis *et al*,^14^ an eQTL analysis was performed using a synovial tissue transcriptomic covariate (PEER factor 2) as an interaction term. To inspect associations between covariates and clinical or histological variables, FDR adjusted p values from linear models for continuous variables, and analysis of variance for categorical variables were calculated. PEER2 exhibited strong correlations with histological pathotype and histological markers CD3, CD20, CD68 and CD138 (online supplemental figure 7A,B). Several SNPs were significant for the interaction PEER2 term using a significance threshold of FDR≤0.05 via a matrixeQTL linear cross model (online supplemental figure 7C). Thus, this analysis identified SNPs where correlation with gene expression varied according to the level of synovial inflammation. Boxplots indicating the adjusted eQTL interaction p values were generated to illustrate this effect for selected significant SNPs and their corresponding gene expression, stratified by pathotype (online supplemental figure 7D–F). Ubiquitination gene, *UBB*, known for its role in immune response,^37^ exhibited significant interaction with PEER2 at the level of both SNP and gene expression (online supplemental figure 7F).

### High association around the HLA complex

**Figure 5:**
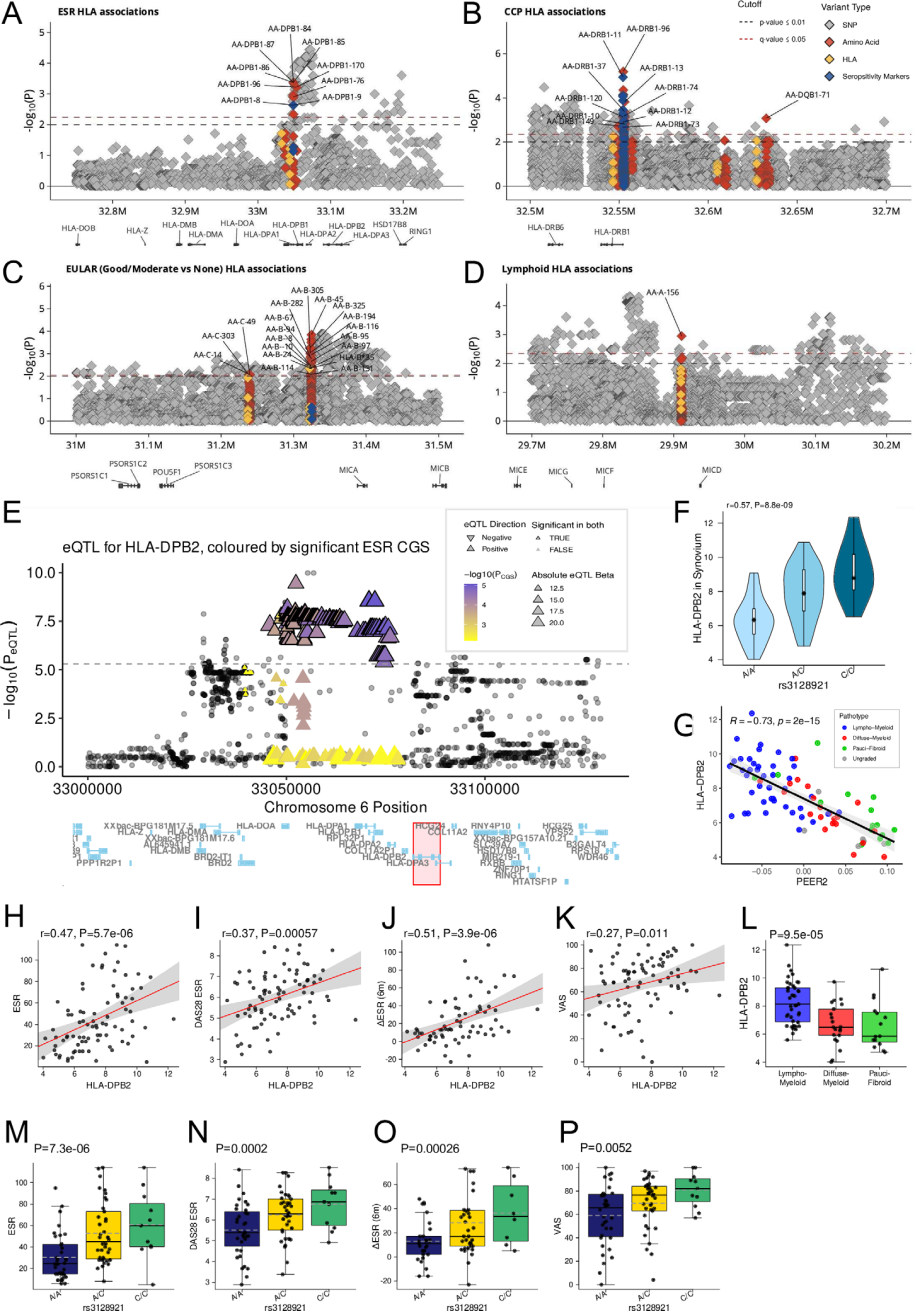
*HLA-DPB2* eQTL is highly associated with disease severity (A–D) Genetic association calculated using linear models via PLINK in HLA region with RA phenotypes (ESR, anti-CCP status, EULAR response and lymphoid subjects). Variants in yellow are imputed HLA residues, those in red are amino acids and those in grey SNPs. Blue markers are the seropositivity markers found by Raychaudhuri *et al* 38. (E) Locus plot for association *HLA-DPB2* eQTL in synovium using MatrixEQTL with statistical analysis by linear regression with four genotype PCA and four PEER factors as covariates. P values were calculated by linear model t-statistic. Significance plotted on the y axis and ESR significant variants are overlaid with colour-coding. Variants significant as both *HLA-DBP2* eSNPS and ESR Candidate Gene Study (CGS) are outlined in black (FDR_eqtl_≤0.01, Q_GWAS_≤0.01, n=234). (F) Genotype vs *HLA-DPB2* expression in synovium for the most significant eQTL variant also significant for ESR (rs3128921). (G–K) Strong correlation (Spearman) between synovial *HLA-DPB2* expression with clinical parameters baseline ESR level, baseline disease activity measured by DAS28-ESR, global Visual Analogue Score (VAS) and clinical response to methotrexate-based DMARD treatment assessed by change in ESR between baseline and 6 months. (M–P) P values calculated via linear model with PCA eigenvectors show association between top SNP rs3128921 with the same disease activity and response clinical variables. DAS28, Disease Activity Score in 28 joints; DMARD, disease-modifying antirheumatic drug; eQTL, expression quantitative trait locu; ESR, erythrocyte sedimentation rate; FDR, false discovery rate; GWAS, Genome-Wide Association Studies.

The strongest genetic associations with RA susceptibility lie with the HLA region and the seminal study by Raychaudhuri *et al* showed that amino acid polymorphisms at *HLA-DRB1*, *HLA-B* and *HLA-DPB1* explain most of the statistical association.^38^ HLA imputation using HLA-TAPAS and linear models via PLINK were employed to assess the association of eQTLs with RA phenotypes in the HLA region. Figure 5A–D displays the eQTLs that showed significant associations (FDR≤0.05), with amino acid-linked SNPs as identified by Raychaudhuri (significantly associated with seropositive RA) highlighted in blue.

This uncovered HLA SNPs associated with histological inflammatory phenotype as defined by the lymphoid pathotype and with systemic inflammation as measured by ESR. Weak associations with EULAR response (good/moderate responders vs non-responders) to methotrexate-based DMARDs at 6 months flagged several variants (eg, HLA-B*35) as possible prognostic markers of response to DMARD treatment. Additionally, multiple variants showed significance following multiple testing correction (FDR≤0.05), replicating results by Raychaudhuri *et al*,^38^ highlighting multiple HLA SNPs and amino acid polymorphisms in particular with association with seropositivity/anticitrullinated protein antibody (ACPA). However, these findings require confirmation in independent data sets due to their moderate significance levels.

To determine whether the significant signals arose from a single, or multiple, association signals, a conditional analysis was performed by accounting for the most significant SNP genotype in a linear model with RA phenotypes (online supplemental figure 8). We concluded that, where there were significant RA phenotype signals (FDR≤0.05) in the HLA region, these were all part of single signals (no conditional q value≤0.05). This analysis was repeated using *HLA-DRB1* shared epitope (SE) associated alleles generated by HLA-TAPAS as covariates, suggesting the effect was not produced by SE alleles (online supplemental figure 9). A full GWAS was performed using linear regression with PLINK to correlate genotype and synovial tissue pathotype on a genome-wide scale (online supplemental figure 10). No significant association was observed. However, the sample size lacks power for analysis of categorical traits.

### HLA-DPB2 shows a triad of correlation between SNPs, cis-gene expression and disease severity

The genotype of significant variants in the HLA region (FDR≤0.05) was correlated to the gene expression of significant cis-eQTL genes in each tissue using a matrixeQTL linear model with covariates. There were 234 *HLA-DPB2* eSNPs which were correlated with *HLA-DPB2* expression in synovial tissue which were also significant for disease activity as measured by serum ESR (figure 5E). Interestingly, *HLA-DPB2* gene expression was also significantly correlated with synovial inflammation as measured by PEER2 and pathotype, with higher levels of *HLA-DPB2* seen in the most inflamed samples with the lympho-myeloid pathotype. *HLA-DPB2* gene expression is also correlated with disease activity as measured by ESR, DAS28, and Visual Analogue Score (VAS) (figure 5G–L), with higher levels of *HLA-DPB2* consistently correlating with more severe disease. Furthermore *HLA-DPB2* expression showed correlation with response to methotrexate-based DMARDs at 6 months in the form of delta ESR from baseline to 6 months, with higher levels of *HLA-DPB2* showing a greater drop in ESR at 6 months. Crucially, rs3128921, a significant *HLA-DPB2* eSNP (figure 5F), was tested for association with disease activity metrics using a linear model with PCA eigenvectors as covariates. This indicated a strong association between the SNP genotype and baseline disease activity metrics ESR, DAS28 and VAS, as well as clinical response to mixed DMARDs as measured by delta ESR from baseline to 6 months (figure 5M–P). This shows an important triad: (1) a set of SNPs at *HLA-DPB2* locus correlate strongly with *HLA-DPB2* expression; (2) *HLA-DPB2* RNA-Seq gene expression also correlates with disease severity and initial response and (3) SNPs at *HLA-DPB2* correlate with disease severity and response. The associations between rs3128921 and baseline ESR and DAS28 as well as response measure delta ESR remained significant after adjusting for *HLA-DRB1* SE alleles (online supplemental table 6), with only the association with patient global VAS no longer reaching significance (p=0.068). None of the SE alleles reached significance (p<0.05) in these linear models, suggesting that the association of rs3128921 is not driven by LD with SE alleles. This evidence supports the notion that variants at the *HLA-DPB2* locus affect RA disease severity and initial response to treatment through an eQTL which affects *HLA-DPB2* expression. However, these results require validation in an independent cohort. *HLA-DPB2* is an HLA pseudogene, but based on publicly available tissue expression data it shows biased expression in lymph node (HPA RNA-seq, ERP003613) and increased expression in dendritic cells and B cells (online supplemental figure 12), consistent with an effect on antigen presentation. GWAS have linked the *HLA-DPB2* locus with other multiple autoimmune diseases including systemic sclerosis,^39^ granulomatosis with polyangiitis,^40^ IgA nephropathy and asthma, as well as age at diagnosis for type 1 diabetes, which would also be consistent with it acting as a risk factor for development of autoimmune disease and as a potential disease severity marker.

## Discussion

This analysis is the only study of synovial tissue eQTL in patients with RA and is one of very few eQTL studies to examine eQTL effects in diseased tissue. The eQTL analysis provides new insight into genetic variations which affect gene expression in synovial tissue, as well as blood, in early RA. Many of the significant eQTL genes and eSNPs have previously been reported to associate with susceptibility to RA and other autoimmune diseases at genome-wide significance levels and thus may give an initial explanation of mechanism for these associations, namely that the susceptibility associated risk SNPs drive altering expression of the linked genes, usually with a cis-eQTL link. This study is the only one to analyse synovial tissue eQTL in patients with RA and among the few to study eQTL effects in diseased tissue. The eQTL analysis sheds light on how genetic variations affect gene expression in synovial tissue and blood in early RA. Several significant eQTL genes and eSNPs have been associated with RA and other autoimmune diseases at genome-wide significance levels, indicating that susceptibility risk SNPs alter gene expression, typically via a cis-eQTL link.

Although there was some overlap of eQTL genes between synovium and blood, the majority were tissue-specific, which was a surprising finding. This tissue specificity was confirmed by comparing the beta values in each tissue. For example, the effect of rs9362226 on NT5E (a gene involved in joint and artery calcification) acted in opposing directions between tissues. This highlights the importance of studying gene expression in disease tissue via biopsies, and demonstrates that inference from blood eQTL studies can lead to erroneous conclusions in terms of the direction of selected genotypic effects. Additionally, several eQTL, such as rs1179623 on TUBB genes, and rs1179623 on HIP1, were significantly different between tissues, indicating that biological effects are captured in only one tissue. Understanding the genomic effects and subsequent pathways involved in each tissue is essential to comprehend the complete picture of the disease.

The eQTL analysis was extended to investigate potential protein QTLs to uncover downstream consequences. Of note, the TNF-inducible gene 6 (TSG-6) pQTL results correlated highly with PEAC synovial *CHST13* eQTL results. This protein has been linked with RA pathogenesis^41^ and has been identified as a biomarker of RA disease activity.^42^ Additionally, previously reported Interleukin-27 pQTL findings from the Genoscores database correlated with *CDC37P1* eQTL associations in PEAC. Previous studies suggest that IL-27 regulates ectopic lymphoid structure formation in RA synovium.^43^ Following protein set enrichment, several histone pathways were found to be significant, which is indicative of an epigenetic effect, and is in keeping with previous reports of epigenetic abnormalities revealed in RA synovial fibroblasts.^35^ Additionally, *IL27* and *IL35* mediated pathways were upregulated, as well as regulation of haemopoiesis. Additionally, *trans-*eQTL analysis highlighted downstream effects between leading *cis-*eSNPs and genes associated with B-cells, osteoblasts and interferons.

Small sample size is a limitation of this study, particularly for trans-eQTL analysis, despite being the largest study to combine synovial tissue gene expression by RNA-Seq with genotype data in matched individuals. Replication of these findings in larger independent cohorts is necessary.

Following a candidate gene analysis of the HLA region, we uncovered an interplay between genotype and a number of RA phenotypes (ESR, CCP, EULAR response and lymphoid subjects). Comparison of these findings with the synovial cis-eQTL results identified multiple synovial eSNPs for *HLA-DPB2* which also significantly correlated with baseline disease activity as measured by ESR, DAS28 and VAS as well as correlating with initial response to mixed DMARD therapy as measured by delta ESR between baseline and 6 months. This shared effect of triangulating genotype, gene expression and inflammation markers is highly suggestive of a clinically important genotypic biomarker for early RA disease severity. However, these findings require validation in an external cohort before top *HLA-DPB2* SNP rs3128921 or *HLA-DPB2* mRNA expression can be thought of as definite prognostic clinical biomarkers.

Overall, our findings illustrate that studying the target tissue of the disease is crucial for identifying the causal biological links between susceptibility genes and development of RA as a disease. Thus, we identify eQTL for known RA susceptibility genes which demonstrates that these SNPs lead to RA susceptibility by altering expression of cis-genes and in some cases transgenes linked to causing RA pathobiology. In multiple cases, eQTL SNPs also showed some degree of association with clinical features including disease severity and response to initial DMARD therapy at 6 months. Our study also identifies additional novel gene variants linked to pathways which mechanistically explain aspects of RA biology and severity, and a particularly strong association was also found for eQTL SNPs at *HLA-DPB2* as a severity marker for RA.

## Materials and methods

### Patients

A total of 144 treatment-naïve patients with RA fulfilling 2010 American College of Rheumatology/European Alliance of Associations for Rheumatology (EULAR) RA Classification Criteria were enrolled at Barts Health National Health Service trust (London, UK) as part of the Medical Research Council (MRC) funded multicentre PEAC (http://www.peac.mrc.mds.qmul.ac.uk) as previously described.^14^ Exclusion criteria included patients receiving corticosteroids, synthetic disease-modifying antirheumatic drug (sDMARDs) or biological therapies.

Synovial tissue samples were obtained through minimally invasive ultrasound-guided biopsy and underwent histological staining and semiquantitative scoring (0–4) for immune cell infiltration using markers for B cells (CD20), T cells (CD3), macrophages (CD68) and plasma cells (CD138). Synovial biopsies were categorised into three synovial pathotypes: lympho-myeloid: diffuse-myeloid and pauci-immune Fibroid, as described previously.^14^

Anonymised baseline radiographs of the hands and feet were scored by a trained reader using the modified Sharp van der Heijde scoring system. Clinical and response data including CRP, ESR, rheumatoid factor (RF), ACPA positivity/titre, patient VAS and DAS28 including the number of tender and swollen joints recorded at baseline (summarised in online supplemental table S1i) and following 6 months of treatment with mixed DMARD therapy (online supplemental table S1ii), which was predominantly methotrexate based (88% of synovium eQTL individuals, 92% of blood eQTL individuals).

### RNA extraction

Sampling and laboratory procedures are described elsewhere.^14^ In short, tissue samples were maintained on ice and homogenised in a fume hood at short 5 s intervals until all the tissue had been sheared/homogenised. The probe of the homogeniser was cleaned between samples using RNase Away solution (Fisher Scientific, UK), followed by four washes in sterile/RNase-free water (Baxter Healthcare, UK).

RNA was extracted from a minimum of 10 mg of synovial tissue homogenised at 4°C in Trizol reagent (ThermoFisher Scientific, Invitrogen Division, UK). Where available, 1 mg of total RNA was used as an input material for library preparation using TruSeq RNA Sample Preparation Kit v2 (Illumina). Generated libraries were amplified with 10 cycles of PCR. Size of the libraries was confirmed using 2200 TapeStation and High Sensitivity D1K screen tape (Agilent Technologies) and their concentration was determined by qPCR based method using Library quantification kit (KAPA). Libraries were multiplexed (five per lane) and then sequenced on Illumina HiSeq2500 to generate 50 million paired-end 75bp reads (154 samples) or 30 million single-end 50bp reads (10 samples).

### RNA-sequencing analysis

Transcript abundance was derived from 154 paired FASTQ files over GENCODE v24/GRCh37 release 87 using STAR 2.7.1a. Transcript counts were imported into R using Bioconductor package GenomicAlignments 1.20.1 providing 137 synovial and 70 blood samples. The RNA-sequencing (RNA-Seq) data underwent conditional quantile normalisation to remove a single systematic effect with length modelled as a smooth function.

The gene expression data were subjected to PEER factor analysis using the PEER package V.1.3 to mitigate the influence of hidden factors that could confound downstream analyses. The data matrix was modelled to account for unmeasured technical variables, batch effects and biological variations. These expression residuals were then employed in subsequent analyses to ensure the robustness of results while minimising the impact of confounding factors. Genes were filtered by expression to a minimum count of 10 and minimum total count of 15. Genes were annotated using the biomaRt package Genome Reference Consortium Human build 37. The RNA-seq data have been deposited in ArrayExpress under Accession code E-MTAB-6141.

### Genotyping

Genomic DNA was isolated from 128 patients with RA using established methods.^44^ Genotyping was carried out using an Illumina Human CoreExome-24 V.1-0 array, following the manufacturer’s protocol and genotypes were called using GenomeStudio (Illumina). Preimputation QC of genotype data excluded SNPs and samples with low call rate (>2% missing). SNPs with <1% minor allele frequency and Hardy Weinberg Equilibrium (p<1×10^−4^) were also removed. Heterozygosity rate of samples were computed and those samples that were 3 SDs away from sample mean were excluded. Identity by descent were computed for all samples to identify duplicate samples or related individuals. Sex checks were performed to identify genetically determined sex mismatches. Following QC, full genotype imputation was prephased with SHAPEIT2 and imputed to 1000 Genomes with IMPUTE2. Post-imputation, SNPs were excluded if they had a postimputation INFO score<0.8 and indels were excluded. SNPs were filtered using minor allele frequency (>0.05), Hardy-Weinberg disequilibrium (p>1×10^−6^), and missing genotype rate (<0.05). The SNP genotypes were encoded according to the number of copies of the minor allele possessed. PCA was conducted on the pruned genotype data (LD≤0.2) to identify covariates using the SNPRelate package (V.1.18.1). GWAS was performed using linear regression with PLINK (V.2.00a) with four genotype PCA as covariates.

### Expression quantitative trait loci analysis

After RNA-sequencing and genotyping, matched data were obtained for 85 synovial and 51 blood samples. The characteristics of these samples are shown in online supplemental table S1). Samples underwent *cis*-eQTL analysis using linear regression models via the MatrixeQTL R package (V.2.3)^29^ (figure 1A). The first four PEER factors from the RNA-seq data and four PCA factors from the genotype data were used as covariates. A genomic cis-window of±0.5 Mb was used centred on each SNP. EQTL were considered to have genome wide significance where FDR≤0.01 using the Benjamini-Hochberg method. In each tissue, genes associated with at least one significant eQTL signal were defined as eQTL genes and the corresponding variants eSNPs. The SNP with the most significant eQTL signal was described as the leading SNP. The matrixEQTL pipeline was subsequently repeated using tissue or PEER2 as an additional covariate in a linear cross model to provide interaction statistics. A linear regression trans-eQTL analysis was also performed on leading cis-eSNPs using matrixeQTL.

Known genome-wide associations for RA, autoimmune diseases and OA were downloaded from the GWAS Catalogue (V.1.0.1).^11^ Significant GWAS risk alleles (p≤5×10^−8^) were compared with eQTL in PEAC. The association between eSNPs and RA response metrics was subsequently calculated using a linear model with four genotype PCA as covariates via the RcppEigen package, similar to the SNPTest methodology. Methods for the Genoscores and eQTL gene enrichment analysis are included in online supplementals.

### Patient and public involvement

Patient and public involvement was led by the MATURA Precision Medicine Patient Advisory Group (chaired by Zoe Ide), who provided continuous support in evaluating trial design, documentation and dissemination of results for the MATURA research programme.

## Data Availability

Data are available in a public, open access repository. The RNA-seq data have been deposited in ArrayExpress under Accession code E-MTAB-6141.
