# Supplementary material for: Expression quantitative trait loci analysis in rheumatoid arthritis identifies tissue specific variants associated with severity and outcome
Source: Ann Rheum Dis. 2023 Nov 18;83(3):288–99. doi: 10.1136/ard-2023-224540 (PMC10894812; doi:10.1136/ard-2023-224540)
Supplement: Supplementary data [file ard-2023-224540supp001.pdf]

Supplementary Methods

Determination of covariates

The number of significant gene-SNP pairs in synovium from matrixEQTL was plotted using varying numbers of genotype PCA eigenvectors and RNA-Seq PEER factors using LD pruning 0.2 and minor allele frequency < 0.05. Using a cut-off for unadjusted p-values of 5×10^{-8} to render simplified direct comparisons, it was identified that 4 eigenvectors and 4 PEER factors were optimal in terms of numbers of significant gene-SNP associations (supplementary figure 11), but without inflating the genomic inflation factor which increased at higher levels of eigenvectors (5 or more) and/or PEER factors (5 or more).

Genomic inflation factor

The genomic inflation factor was calculated defined as the median of the observed chi-squared test statistic divided by the expected median of the corresponding chi-squared distribution. For synovial samples the genomic inflation factor was λ_{gc}=1.02 and in blood λ_{gc}=1.04 using 4 genotype eigenvectors and 4 PEER factors.

Imputation of the Human Leukocyte Antigen (HLA) System

Using methods similar to Raychaudhuri et al. [1], amino acid polymorphisms and single nucleotide polymorphisms (SNPs) in the HLA system within the major histocompatibility complex (MHC) region in chromosome 6 were imputed using HLA-TAPAS [2] with a reference panel built from 2,504 individuals from the 1000 Genomes project [3].

PLINK (v2.00a) was used for a candidate gene study in the HLA region for 128 RA samples, incorporating the first four principal component eigenvalues as covariates. Ten subjects were removed following PLINK filtering. We used CCP, CRP, CD20, VAS, ESR, Krenn inflammatory score, pathotype, $DAS28_{ESR}$, change in $DAS28_{ESR}$, and EULAR response as phenotypes. PLINK uses linear regression models with t-statistic to calculate p-values. In cases of multiple

causal eSNPs, a conditional analysis was performed to identify independent eSNPs for each significant eQTL gene by accounting for the genotype of the leading SNP in a linear model.

Enrichment of eQTL genes

Enrichment of transcription factors and disease pathways among eQTL genes in each tissue was conducted using the topGO R package (v2.48) [4].

Genoscores Analysis

The GENOSCORES platform (https://genoscores.cphs.mvm.ed.ac.uk/) [5, 6] was used to compute genotypic scores in the PEAC cohort for loci with significant cis-eQTL signals in synovial gene expression (eQTL scores). We also computed locus-specific genotypic scores for 1,478 proteins circulating in plasma using publicly available protein QTL summary statistics, downloaded from the INTERVAL study [7]. Levels of 3,622 proteins circulating in plasma were measured using the SOMAscan assay in 3,301 healthy participants identifying 1,927 protein QTL for 1,478 proteins. Full summary statistics for these proteins were imported into the GENOSCORES database and used in the present analysis along with the summary statistics for the detected synovial cis-eQTLs.

To compute locus-specific genotypic scores, we clumped trait-associated SNPs around each lead QTL ensuring at least 1Mb gap between such clumps. A score was then computed for each clump by multiplying the matrix of target genotypes by a vector of SNP effect sizes from eQTL or pQTL summary statistics. The vector of SNP effect sizes was pre-adjusted for linkage disequilibrium by multiplying with the inverse of a SNP-SNP correlation matrix computed in the European ancestry subset of the 1000 Genomes panel [8]. Locus-specific pQTL scores were classified as cis, where the pQTL score was within 50kb from the transcription site of the corresponding gene, and trans, where the pQTL score was more than 50kb away from the transcription site. Proteins were mapped to genes using their uniprot IDs.

To determine if the same underlying haplotypes drive the signals for gene expression and protein levels, we computed genetic correlations between eQTL and pQTL scores located

within 200kb from each other. eQTL-pQTL score-score correlations were calculated as the correlation between eQTL and pQTL correlation scores within a locus window. An eQTL-pQTL score-score correlation with an r value above 0.5 was used as suggestive evidence of a shared genetic signal for synovial mRNA expression and for protein level with the same direction of effect. For proteins that had an eQTL-pQTL score-score correlation above 0.5, enrichment analysis was conducted using the UniProt.ws R package (v2.35).

Resources and Software packages

The following software packages were used in analysis:

- GENCODE v24/GRCh37 release 87 [9]
- STAR v2.7.1a [10]
- GenomicAlignments v1.20.1 [11]
- PLINK v2.0 [12]
- SNPRelate package v1.18.1 [13]
- PEER package v1.3 [14]
- MatrixeQTL v2.3 [15]
- HLA-TAPAS [2]
- topGo v2.52 [4]
- UniProt.ws R package v2.35 [16]
- Genoscores platform [5, 6]

Supplement References

1. Raychaudhuri, S., et al., *Five amino acids in three HLA proteins explain most of the association between MHC and seropositive rheumatoid arthritis.* Nat Genet, 2012. **44**(3): p. 291-6.
2. Luo, Y., et al., *A high-resolution HLA reference panel capturing global population diversity enables multi-ethnic fine-mapping in HIV host response.* medRxiv, 2020.
3. The 1000 Genomes Project Consortium, et al., *A global reference for human genetic variation.* Nature, 2015. **526**(7571): p. 68-74.
4. Alexa, A. and J. Rahnenfuhrer, *topGO: Enrichment Analysis for Gene Ontology*. 2022. p. R package.
5. Spiliopoulou, A., et al., *Association of response to TNF inhibitors in rheumatoid arthritis with quantitative trait loci for CD40 and CD39.* Ann Rheum Dis, 2019. **78**(8): p. 1055-1061.
6. McKeigue, P., et al. *Genoscores: a platform for calculating genotypic predictors of binary and quantitative phenotypes*. 2020; Available from: https://genoscores.cphs.mvm.ed.ac.uk/.
7. Sun, B.B., et al., *Genomic atlas of the human plasma proteome.* Nature, 2018. **558**(7708): p. 73-79.

8. Clarke, L., et al., *The international Genome sample resource (IGSR): A worldwide collection of genome variation incorporating the 1000 Genomes Project data.* Nucleic Acids Res, 2017. **45**(D1): p. D854-D859.
9. Flicek, P., et al., *Ensembl 2014.* Nucleic Acids Res, 2014. **42**(Database issue): p. D749-55.
10. Dobin, A., et al., *STAR: ultrafast universal RNA-seq aligner.* Bioinformatics, 2013. **29**(1): p. 15-21.
11. Lawrence, M., et al., *Software for computing and annotating genomic ranges.* PLoS Comput Biol, 2013. **9**(8): p. e1003118.
12. Chang, C.C., et al., *Second-generation PLINK: rising to the challenge of larger and richer datasets.* Gigascience, 2015. **4**: p. 7.
13. Zheng, X., et al., *A high-performance computing toolset for relatedness and principal component analysis of SNP data.* Bioinformatics, 2012. **28**(24): p. 3326-8.
14. Stegle, O., et al., *Using probabilistic estimation of expression residuals (PEER) to obtain increased power and interpretability of gene expression analyses.* Nature protocols, 2012. **7**(3): p. 500-507.
15. Shabalin, A.A., *Matrix eQTL: ultra fast eQTL analysis via large matrix operations.* Bioinformatics, 2012. **28**(10): p. 1353-8.
16. Carlson, M., *UniProt.ws: R Interface to UniProt Web Services*. 2021, Bioconductor: https://bioconductor.org/packages/release/bioc/html/UniProt.ws.html.

Figure S1: Covariate analysis of genotype and RNA-seq data

(A) Principal component analysis on genotype samples show clustering of ethnicity in both the 1000 Genome reference panel (left) and PEAC (right). (B) PEER factors analysis in Blood RNA-seq data indicates a batch effect.

A

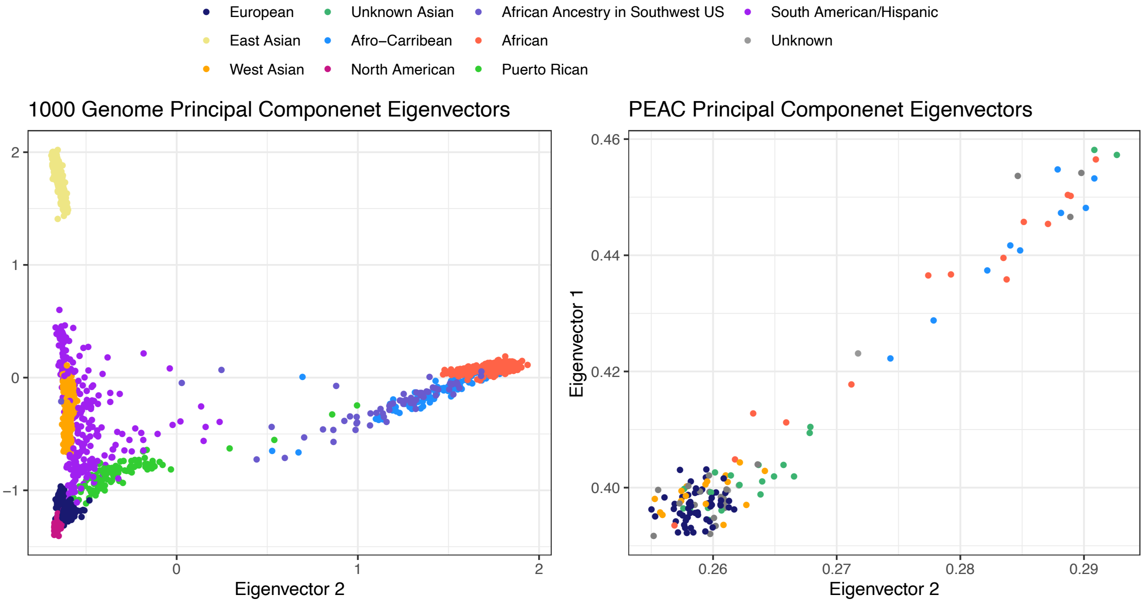

B

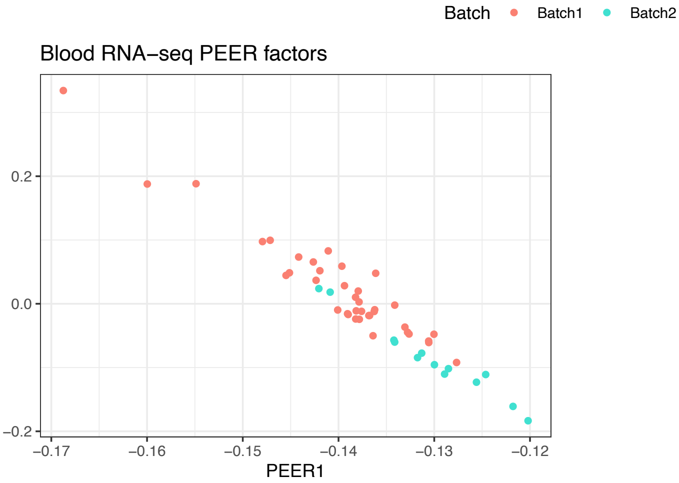

Figure S2: Locus plots for the 28 genes and SNPs which are significant in synovium and have significant eSNPs with known RA, OA, or autoimmune association in GWAS catalog

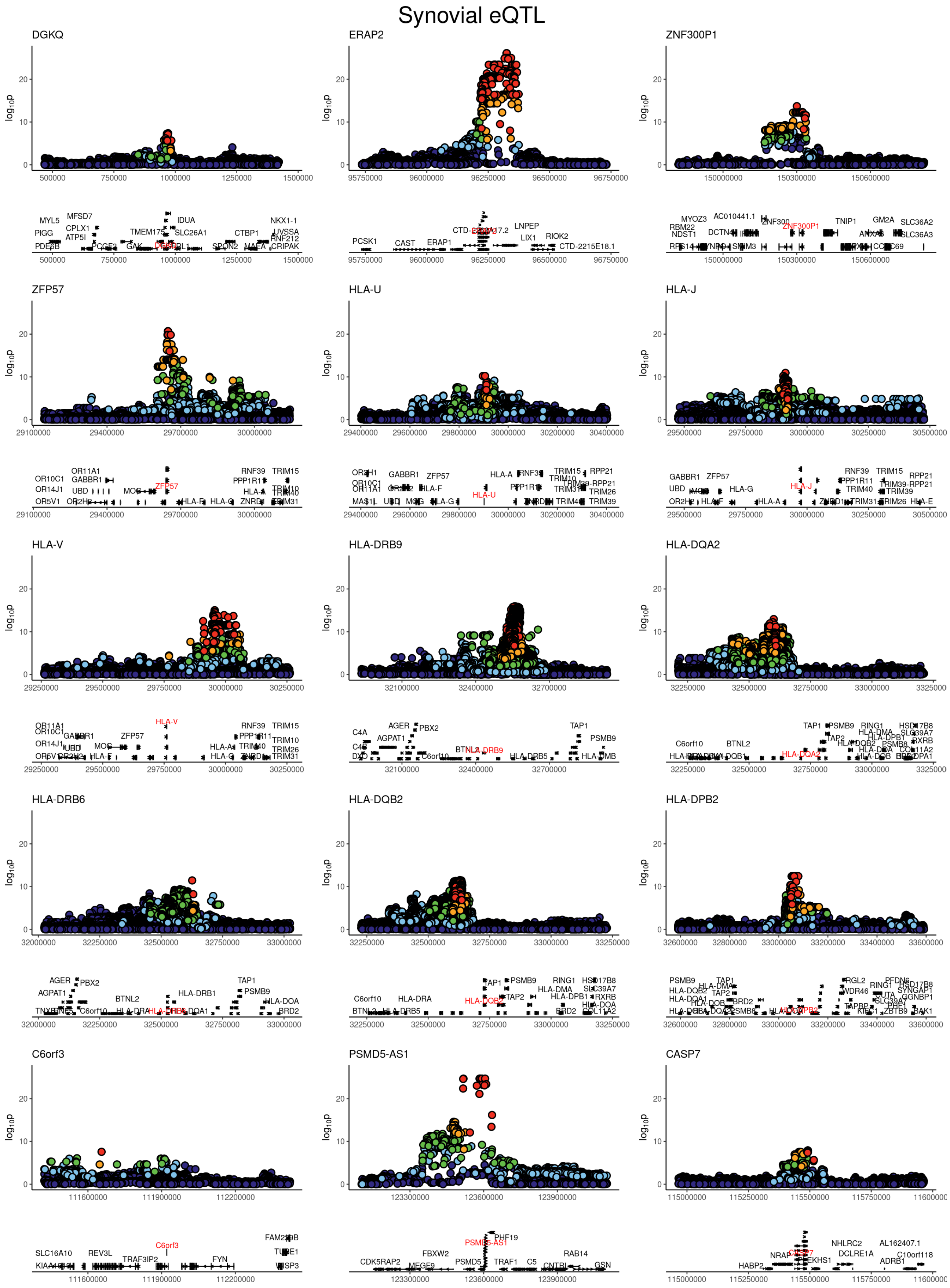

Synovial eQTL continued

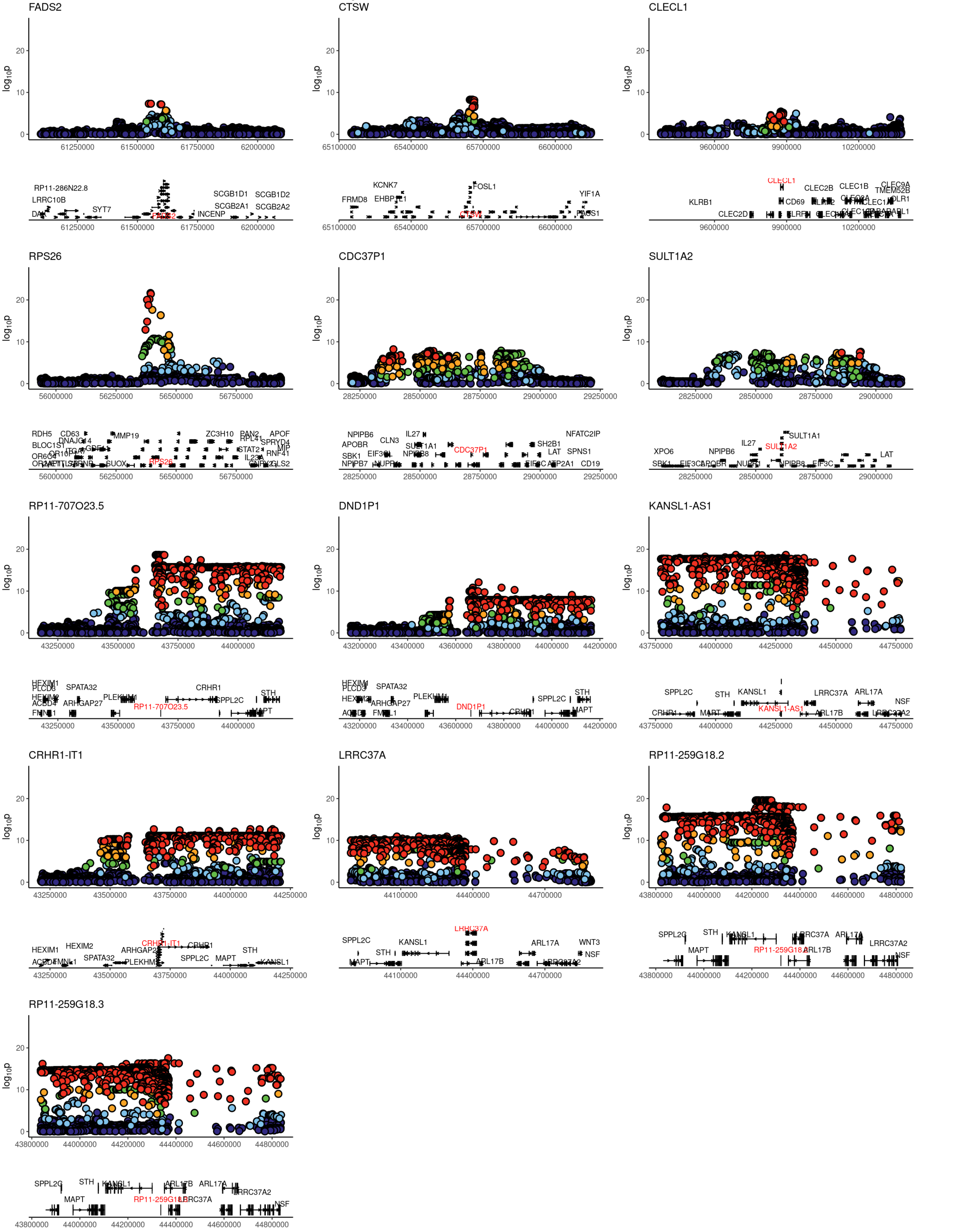

Figure S3: Locus plots for the 39 genes and SNPs which are significant in blood and have significant eSNPs with known RA, OA, or autoimmune association in GWAS catalog

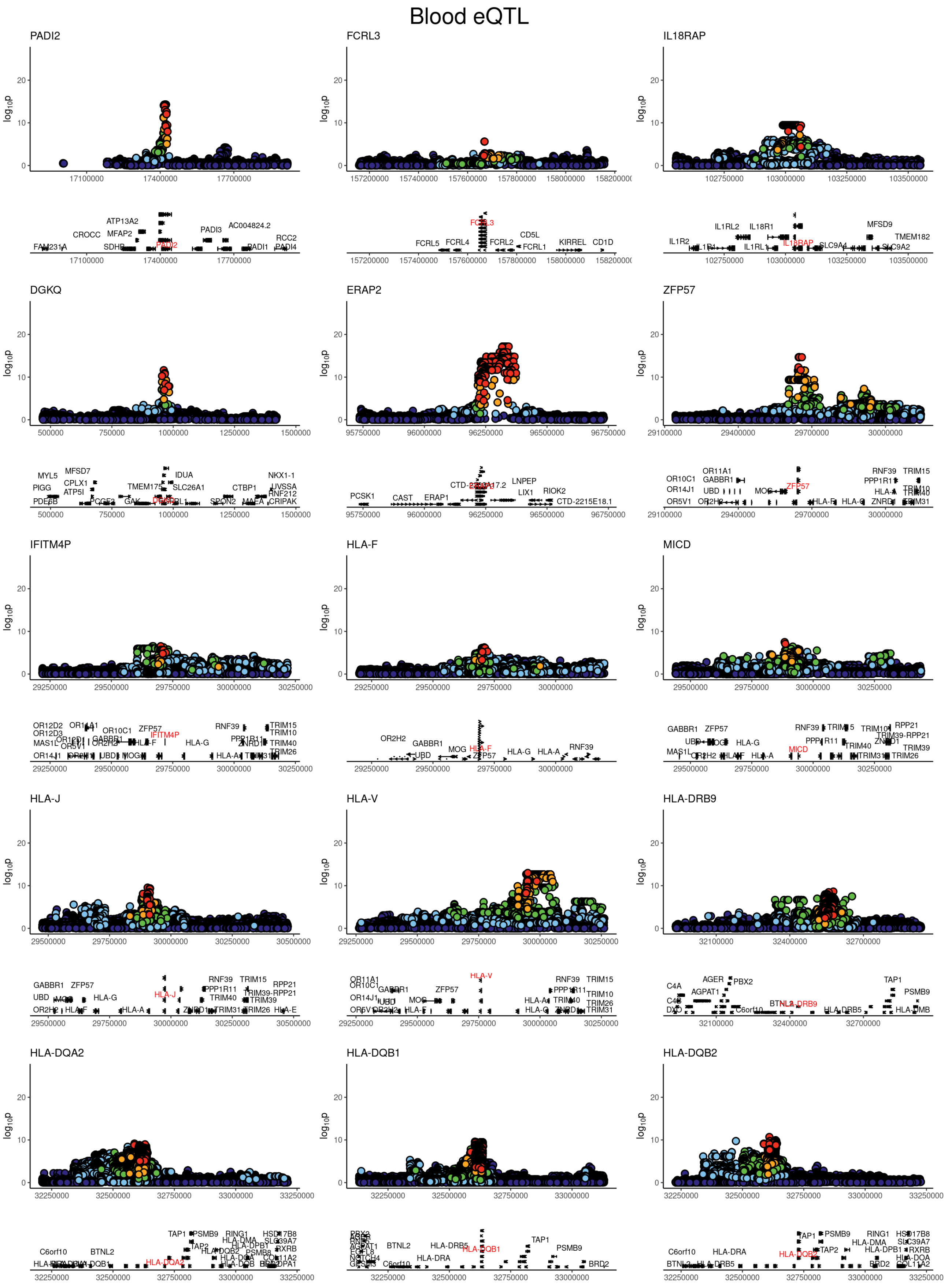

Blood eQTL continued

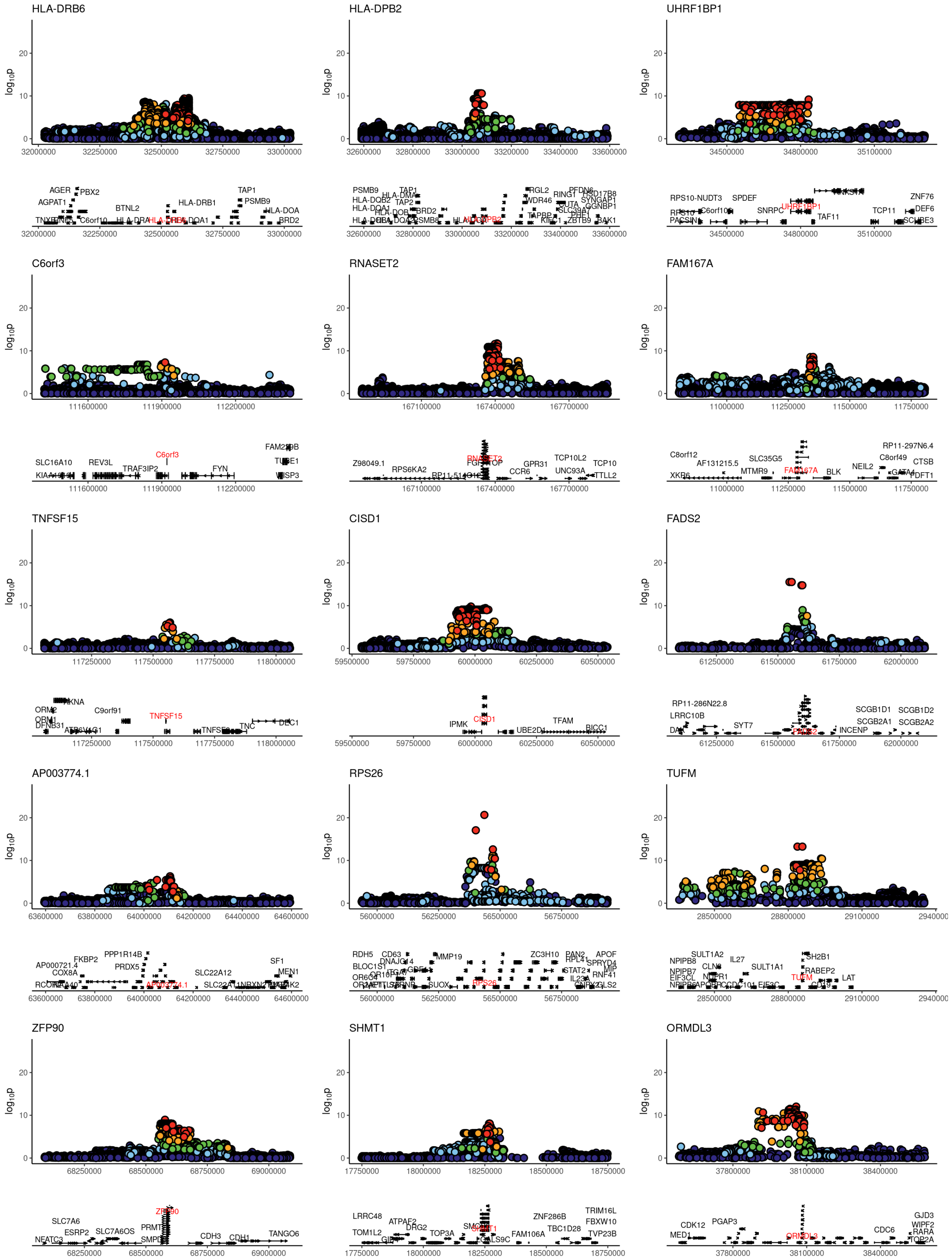

Blood eQTL continued

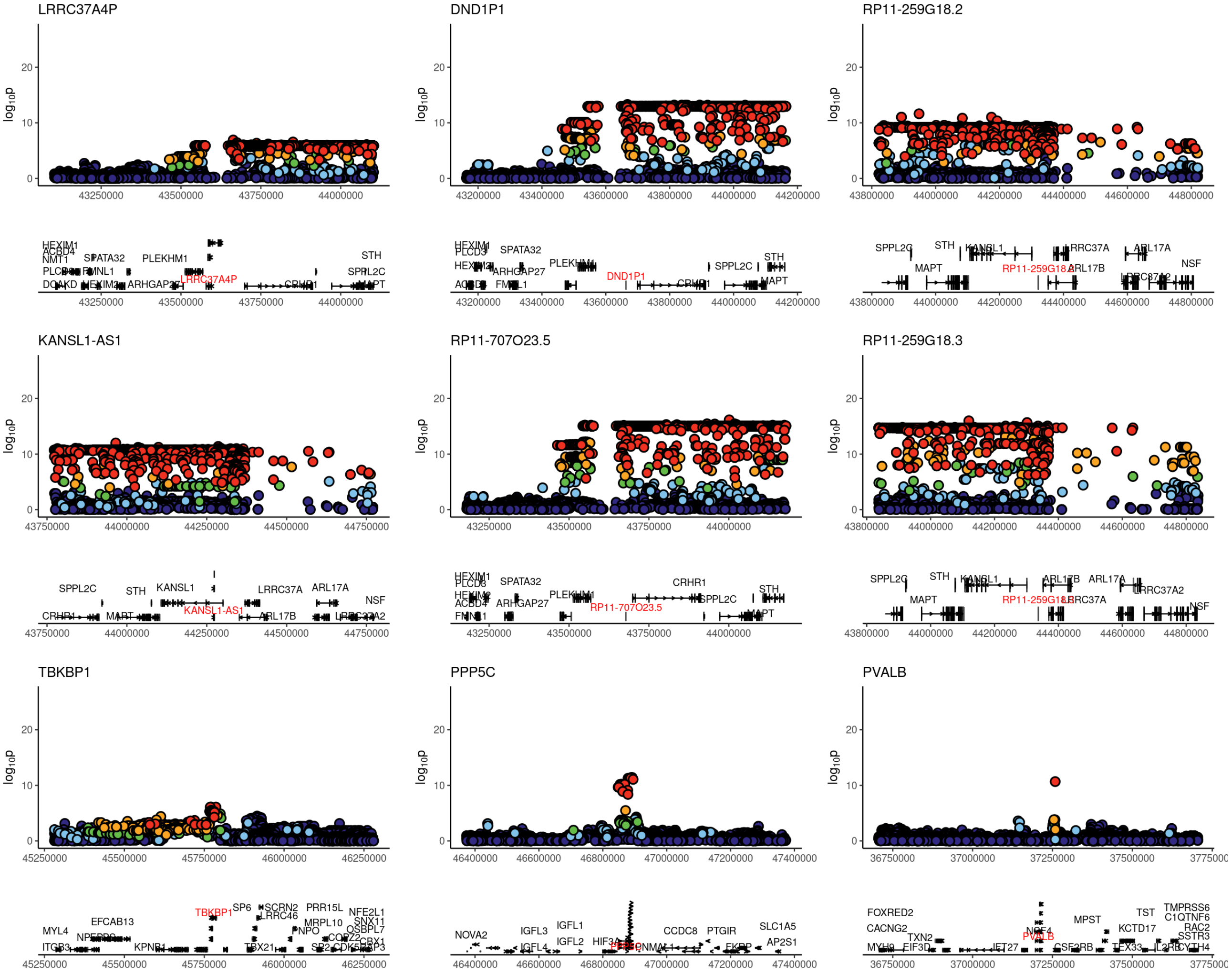

Figure S4: Enrichment of gene ontologies using significant eGenes where p-value arises from a Kolmogorov-Smirnov test

Abbreviations: BP: Biological Process, CC: Cellular Component, MF: Molecular Function.

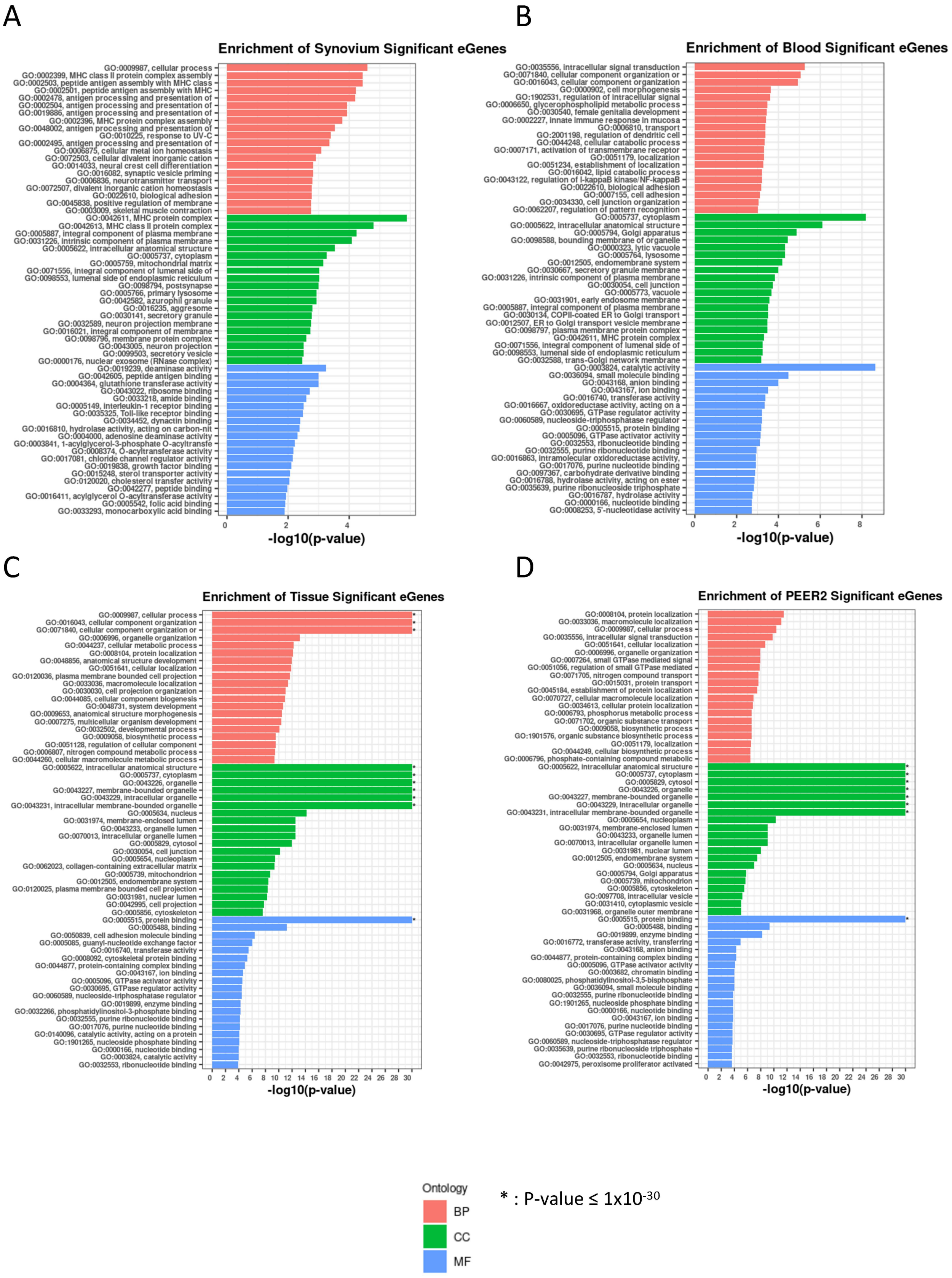

Figure S5: Pathway enrichment with UniprotR for genoscores proteins

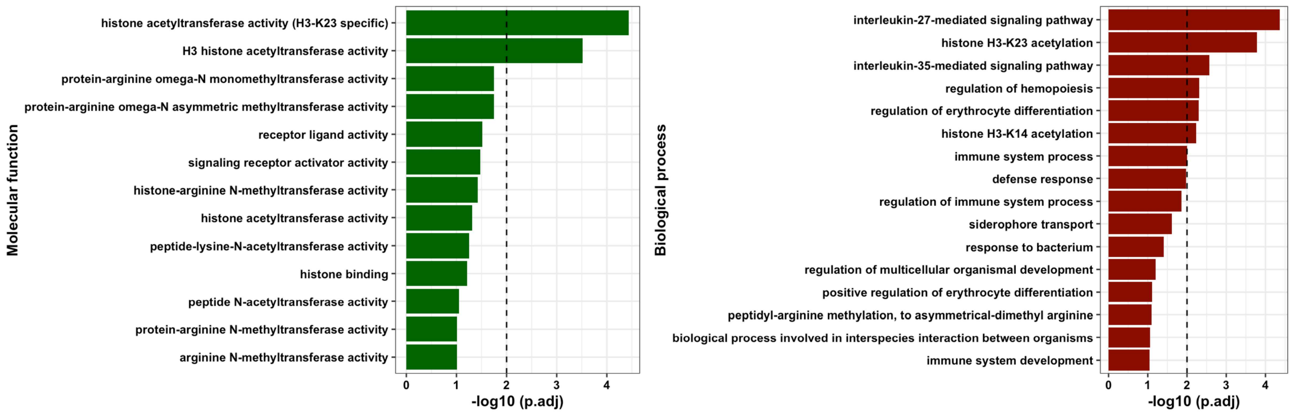

Figure S6: eQTL circus plots for linear regression trans effects of the leading *cis*-eSNP for (A) *CXCL9* and (B) *DAPK1*

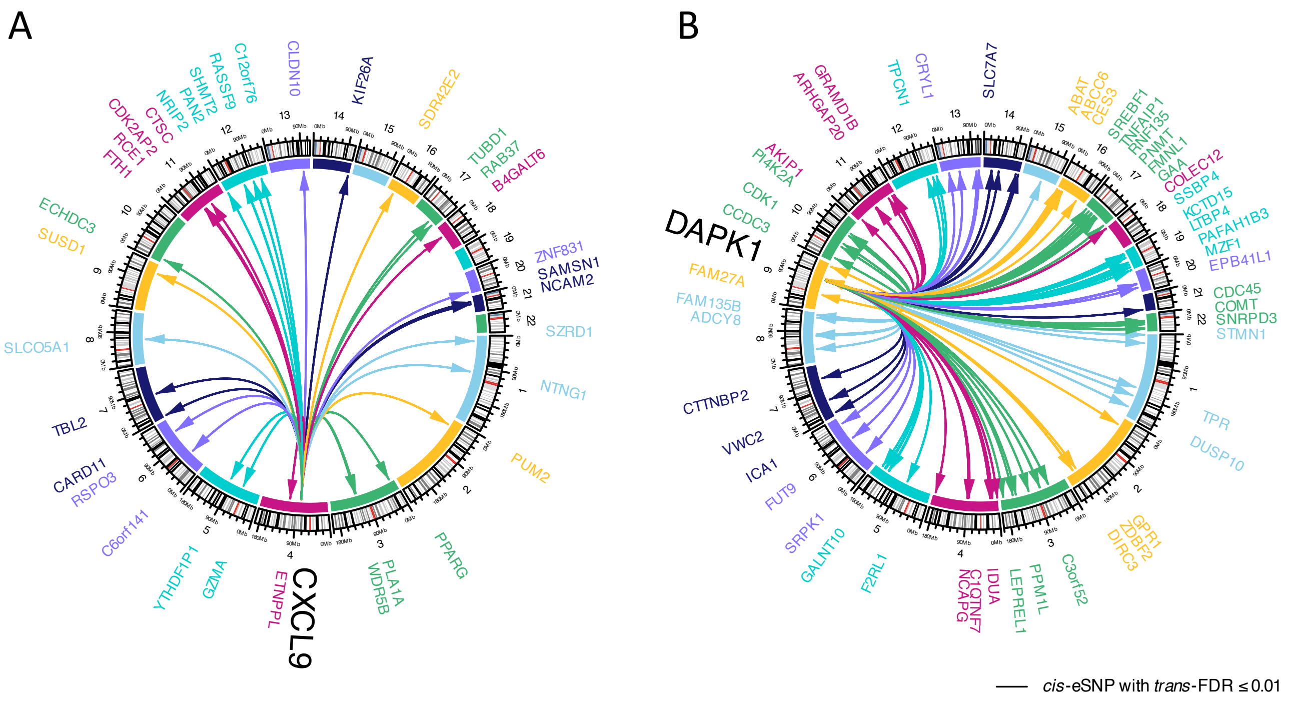

Figure S7: Interaction between PEER2 and eQTL effects

(A) Associations between the covariates (principal component eigenvalues, EV1-4, and PEER factors) with clinical or response variables (q-values indicate FDR adjusted p-values from linear model for continuous variables and ANOVA for categorical variables). Correlations are outlined where significant (q ≤ 0.05). (B) PEER factor plot in synovial samples emphasising the pathotype effect. (C) SNP-PEER2 interactions from eQTL linear cross model, where the dashed line reflects FDR ≤ 0.05. (D-F) Boxplots for a selection of synovial gene expression versus genotype with subject pathotype coded by colour.

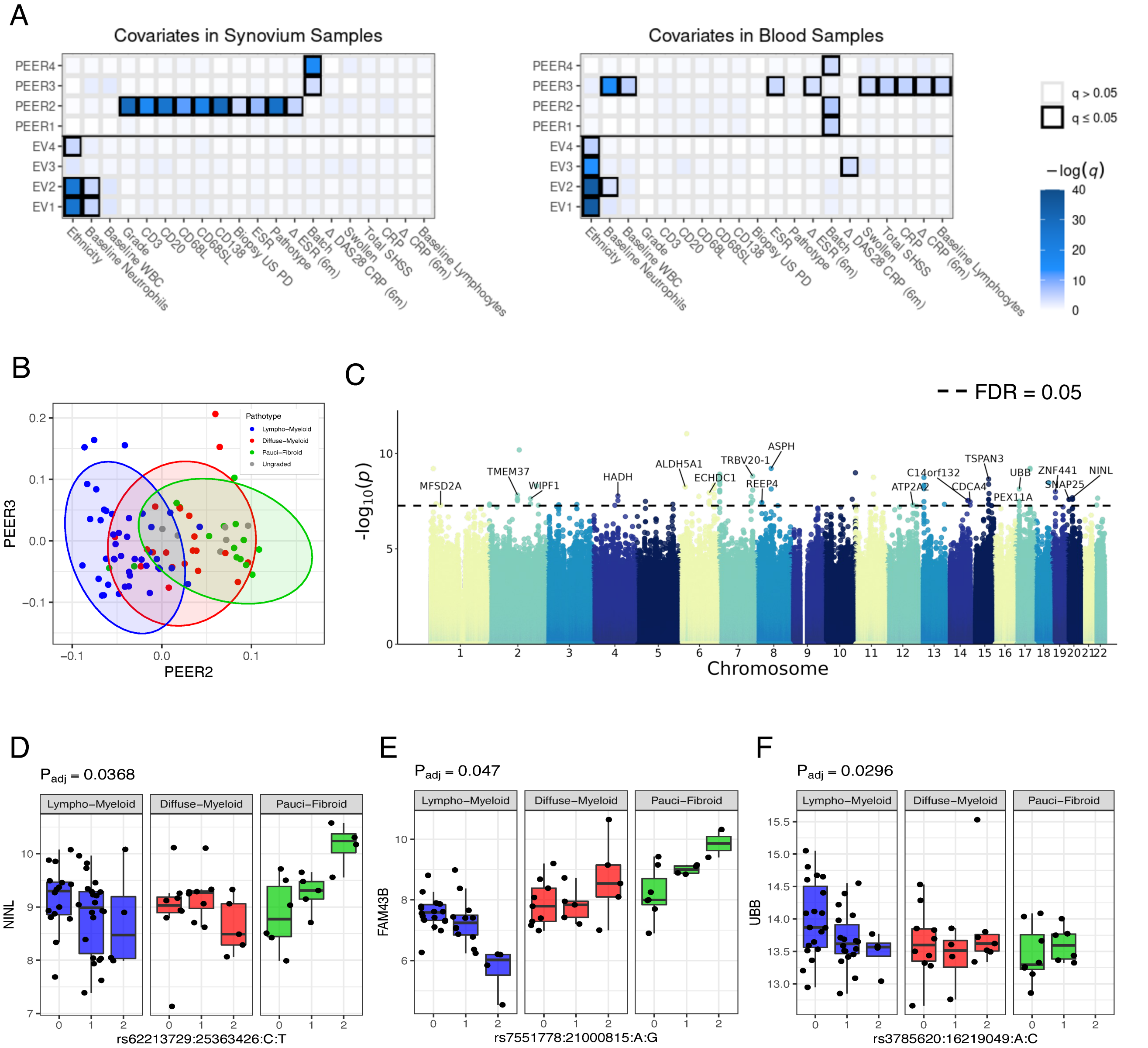

Figure S8: Conditional association between RA phenotypes and imputed HLA alleles in the MHC region

Upper panels (blue) show association between imputed HLA alleles and phenotypes using a linear model with Plink. Lower panels (orange) represent the conditional association given a linear model which features the genotype for the most significant SNP as an additional covariate. This shows for each phenotype there is never more than one significant signal.

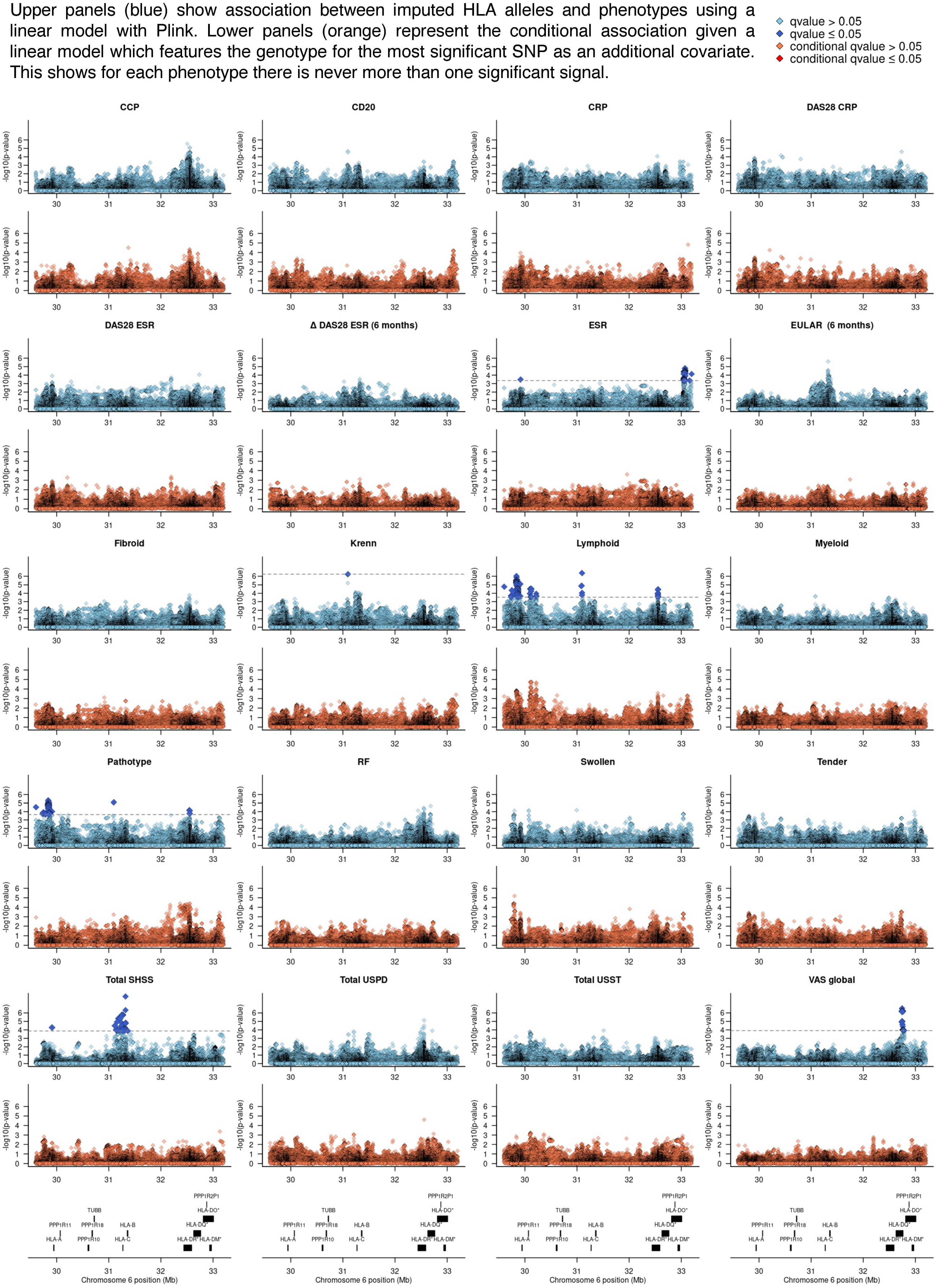

Figure S9: Conditional association between RA phenotypes and imputed HLA alleles in the MHC region correcting for shared epitope *HLA-DRB1* alleles

Association between imputed HLA alleles and phenotypes using a linear model with PLINK with PCA eigenvectors and *HLA-DRB1* SE alleles as covariates.

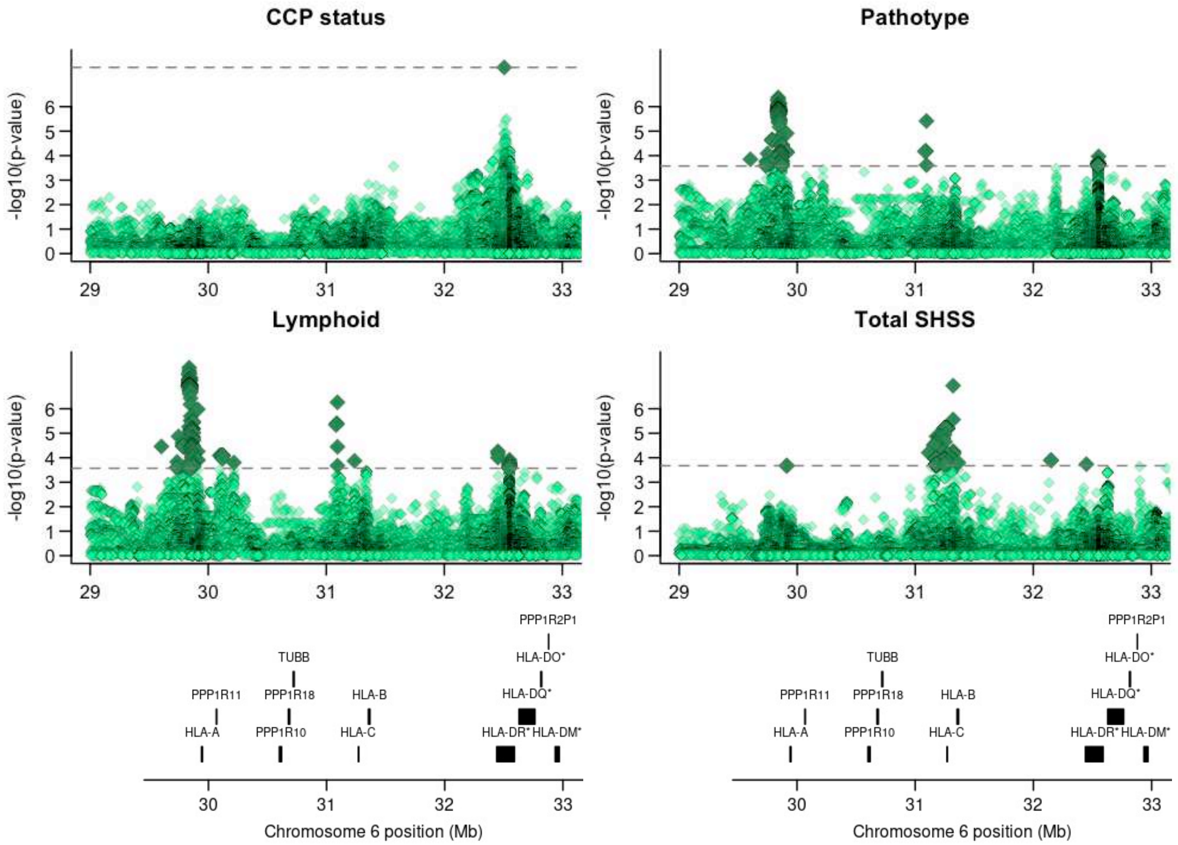

Figure S10: GWAS results for pathotype using linear regression with PLINK

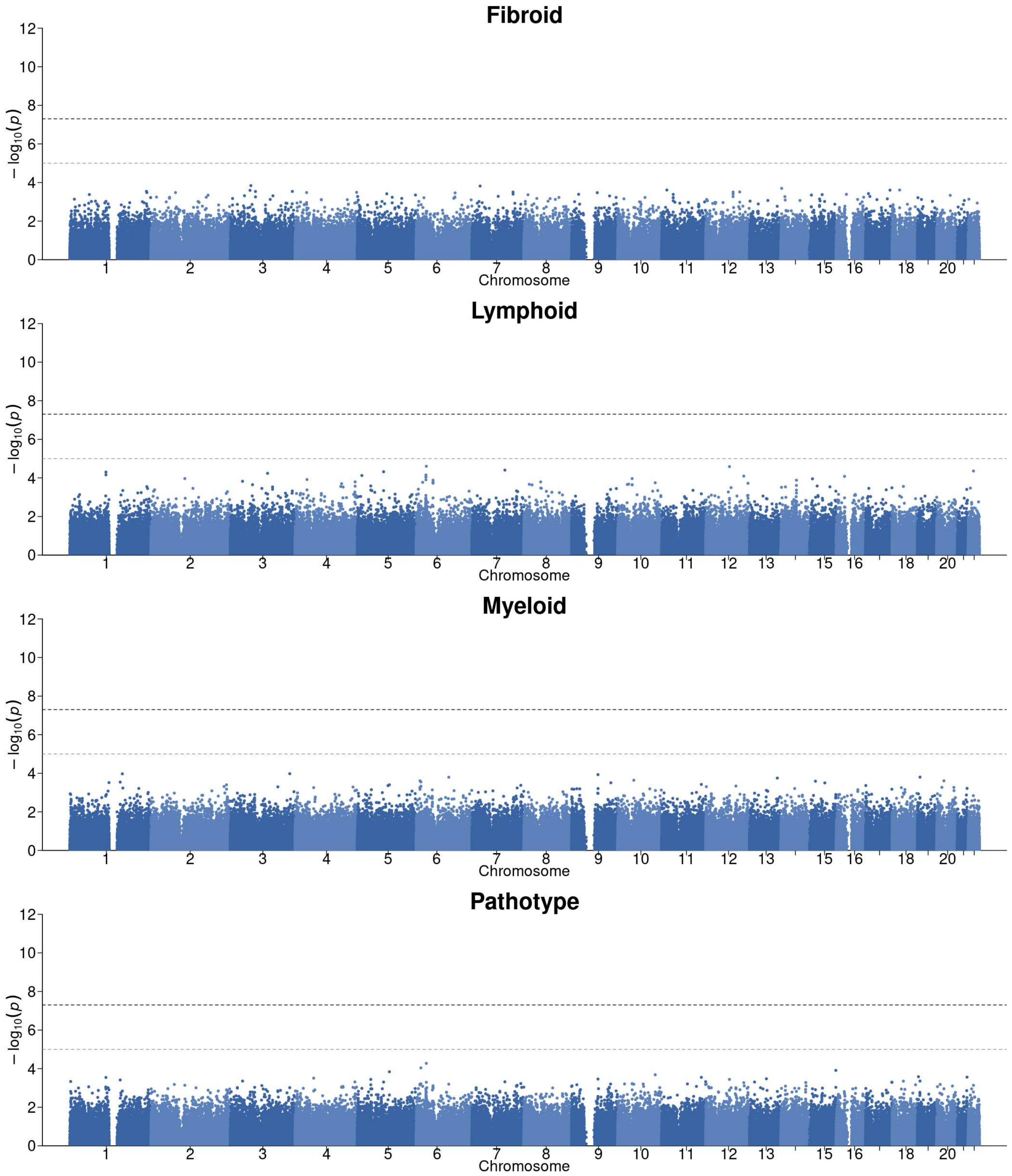

Figure S11: The number of significant synovial eQTL reaching genome-wide significance using varying numbers of covariates

The number of significant gene-SNP pairs in synovium from matrixEQTL using different numbers of PCA eigenvectors (*x*-axis) or PEER factors (grid) using LD pruning 0.2 and minor allele frequency < 0.05.To render direct comparisons an unadjusted p-value cutoff of 5 x 10^{-8} was employed throughout.

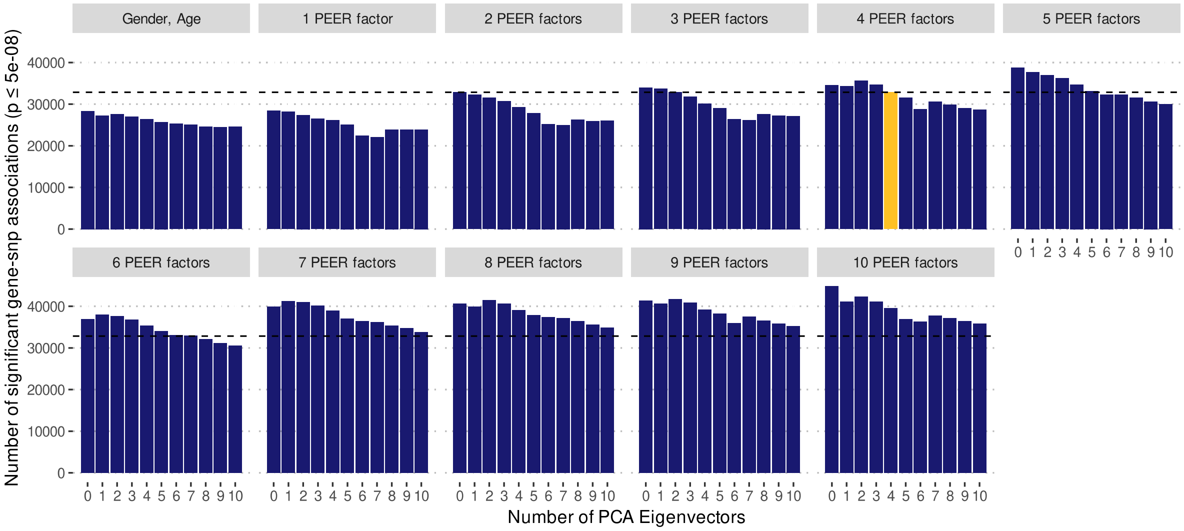

Figure S12: Expression of HLA-DPB2 in FANTOM5 tissue repository

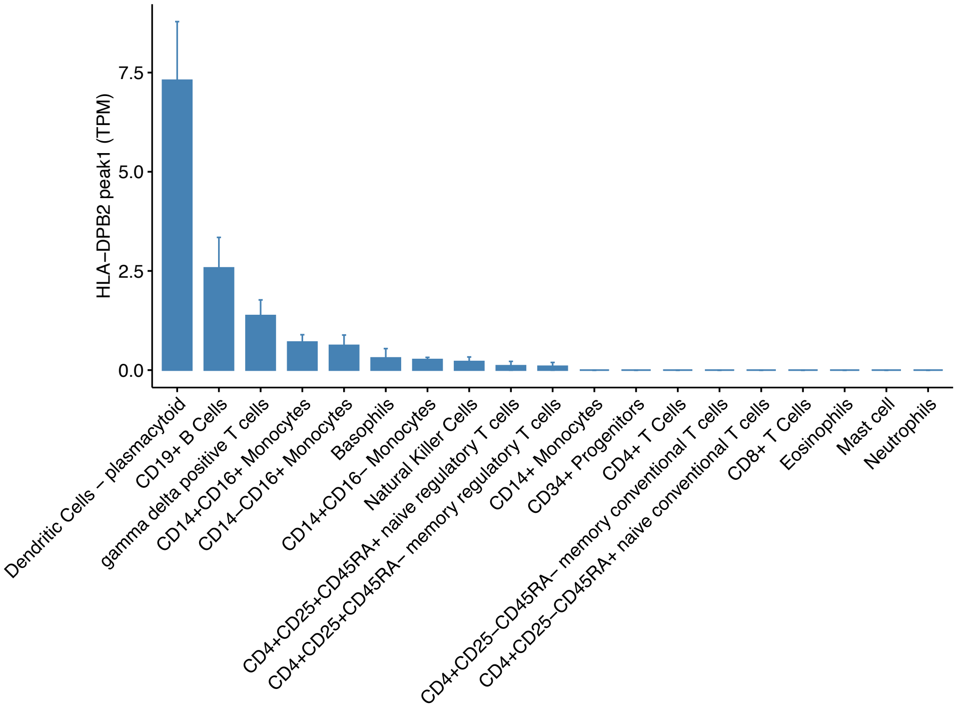

Table S1: Pathobiology of Early Arthritis Cohort (PEAC) demographics (i) characteristics and (ii) treatment

i)

	Genotyped Samples	Synovial eQTL Samples	Blood eQTL Samples	Overlapping Samples
n	118	85	51	45
Sex, % female	71	74	67	69
EULAR, % good responder	43	46	43	41
CCP, % positive	65	71	67	69
Age	50 (38-61)	52 (43-61)	54 (42-66)	54 (44-67)
CCP	213 (2-340)	224 (2.6-340)	239 (2.5-522)	246 (3-600)
RF	120 (15-137)	136 (16-169)	111 (15-150)	119 (15-158)
CRP	20 (5-22)	24 (5-33)	25 (5-34)	27 (5-36)
ESR	39 (16-51)	44 (24-61)	44 (27-59)	46 (28-65)
Tender Joint Count	12 (6-16)	12 (7-17)	12 (5.5-17)	13 (6-19)
Swollen Joint Count	7.5 (4-9)	7.9 (4-9)	8.7 (4-12)	8.8 (4-12)
DAS_{28}	5.7 (5-6.8)	6 (5.2-6.8)	6 (5.1-7)	6.1 (5.1-7)
ΔDAS_{28}	2 (0.92-3.2)	2.1 (1-3.3)	2.1 (0.97-3.4)	2.2 (0.95-3.5)

ii)

Treatment	Synovium eQTL individuals	Blood eQTL individuals
Methotrexate/ Sulfasalazine	50	23
Methotrexate/ Hydroxychloroquine	16	15
Methotrexate/ Sulfasalazine/ Hydroxychloroquine	5	5
Methotrexate	4	4
Hydroxychloroquine	6	2
Sulfasalazine	3	1

Supplementary tables S2 and S3 can be found in Tables_S2_and_S3.xlsx

Table S2: Significant (FDR ≤ 0.01) eGenes for synovium.
Table S3: Significant (FDR ≤ 0.01) eGenes for blood.

Table S4: Significant correlations (FDR ≤ 0.05) of eSNPs with RA response variables

Leading SNP	eQTL Gene	Number of significant variants	Response variable	Beta	SE	P-value	Q-value
Synovium							
rs34492166	RNASEH1-AS1	16	Delta CRP (six-months)	31.80	6.39	7.4e-06	7.0e-03
rs6876611	ERAP2	180	Delta ESR (1 year)	-15.41	4.16	4.7e-04	3.7e-02
rs2104616	PEX6	50	Delta ESR (six-months)	15.47	3.67	7.2e-05	4.3e-02
rs6477887	SUSD1	14	Delta DAS28 CRP (six-months)	1.03	0.26	1.7e-04	1.7e-02
rs41278323	SLC25A51	7	Delta DAS28 CRP (six-months)	1.34	0.35	3.0e-04	1.7e-02
rs41278323	SLC25A51	7	Delta DAS28 CRP (1 year)	1.89	0.37	3.3e-06	2.5e-04
rs41278323	SLC25A51	7	Delta DAS28 ESR (1 year)	2.18	0.42	2.5e-06	1.9e-04
rs113154888	DCAF10	1	Delta DAS28 CRP (six-months)	1.26	0.32	2.0e-04	1.7e-02
rs113154888	DCAF10	1	Delta DAS28 ESR (1 year)	1.79	0.40	3.4e-05	2.0e-03
rs113154888	DCAF10	1	Delta DAS28 CRP (1 year)	1.62	0.35	1.6e-05	9.2e-04
rs5017041	BTBD16	19	Delta DAS28 ESR (1 year)	1.11	0.28	1.9e-04	4.2e-02
rs67260198	RP5-1021I20.1	21	Delta DAS28 CRP (1 year)	1.37	0.36	3.2e-04	2.8e-02
rs67260198	RP5-1021I20.1	21	Delta DAS28 ESR (1 year)	1.70	0.41	1.1e-04	9.8e-03
rs394866	FAHD1	153	Delta DAS28 CRP (six-months)	1.35	0.37	6.0e-04	1.2e-02
rs9940552	SPIRE2	37	Delta DAS28 CRP (six-months)	0.74	0.23	2.3e-03	1.8e-02
Blood							
rs35508555	CCDC146	49	Delta CRP (six-months)	45.44	7.78	9.3e-07	3.1e-03
12:8206167:A:G	C3AR1	9	Delta DAS28 ESR (1 year)	1.82	0.35	1.1e-05	1.7e-02
12:8206167:A:G	C3AR1	3	Delta DAS28 CRP (1 year)	1.56	0.30	1.1e-05	1.7e-02
rs1341486	SPG20	176	Delta ESR (1 year)	28.88	6.96	1.9e-04	6.6e-03
rs12887106	RP5-1021I20.1	147	Delta DAS28 ESR (1 year)	1.57	0.44	1.1e-03	9.5e-03
rs12887106	RP5-1021I20.1	123	Delta DAS28 CRP (1 year)	1.26	0.41	4.0e-03	4.0e-02
rs71421237	BEGAIN	1	Delta DAS28 ESR (1 year)	1.21	0.39	4.1e-03	1.7e-02
rs12439805	MAN2A2	19	Delta ESR (six-months)	29.79	6.54	3.7e-05	2.1e-02
rs1984357	LMAN1	10	Delta ESR (1 year)	22.44	5.73	3.6e-04	1.8e-02

Table S5: Genoscores analysis showing the correlations between locus-specific genotypic scores for synovial gene expression and locus-specific genotypic scores for plasma protein levels

Gene	Chromosome	Correlation	Protein	Trans - Protein QTL chromosome	Diseases protein is associated with
KLHL7-AS1	7	-0.999	Transmembrane glycoprotein NMB	7	Amyloidosis, primary localized cutaneous, 3 (PLCA3) [MIM:617920]
KLHL7-AS1	7	-0.782			
SPSB2	12	-0.998	Transketolase	3	Short stature, developmental delay, and congenital heart defects (SDDHD) [MIM:617044]
LRRC23	12	-0.995			
CHST13	3	-0.944	Tumor necrosis factor-inducible gene 6 protein	6	Autoinflammatory syndrome, familial, Behcet-like (AISBL) [MIM:616744]
CDC37P1	16	-0.94	Interleukin-27	2	Immunodeficiency 31B (IMD31B) [MIM:613796]; Immunodeficiency 31A (IMD31A) [MIM:614892]; Immunodeficiency 31C (IMD31C) [MIM:614162]
GSDMA	17	-0.93	Resistin	8	
CHST13	3	-0.928	NAD-dependent protein deacetylase sirtuin-2	19	
RP11-996F15.2	12	0.928	GDH/6PGL endoplasmic bifunctional protein	1	Cortisone reductase deficiency 1 (CORTRD1) [MIM:604931]
GSDMA	17	-0.906	Neutrophil gelatinase-associated lipocalin	14	
WDR66	12	-0.899	Peregrin	3	Intellectual developmental disorder with dysmorphic facies and ptosis (IDDDFP) [MIM:617333]
HLA-DQA2	6	0.88	Aurora kinase B	17	
HLA-DQB2	6	0.844			
PEX6	6	-0.844	Glycine N-methyltransferase	19	Cerebral creatine deficiency syndrome 2 (CCDS2) [MIM:612736]
NT5C3B	17	-0.835	2',3'-cyclic-nucleotide 3'-phosphodiesterase	17	
HCG4	6	0.792	Ameloblastin	4	Amelogenesis imperfecta 1F (AI1F) [MIM:616270]
HCG4P5	6	0.772			
HLA-K	6	0.754			
PILRB	7	0.79	Histatin-1	4	
HLA-DRB9	6	0.783	GDNF family receptor alpha-2	8	
HLA-DQB2	6	0.733			
CHST13	3	-0.77	Inter-alpha-trypsin inhibitor heavy chain H1	3	
SIGLEC12	19	0.754	Sialic acid-binding Ig-like lectin 12	20	
PEX6	6	0.752	Mediator of RNA polymerase II transcription subunit 1	17	
GSDMA	17	-0.743	AT-rich interactive domain-containing protein 3A	19	
HLA-K	6	-0.736	Layilin	11	
HLA-K	6	-0.727	Tapasin-related protein	12	

Tables S6: Linear models indicating clinical variables with rs3128921 and shared epitope alleles and PCA eigenvectors.

ESR ~ rs3128921 + HLA_DRB1*01 + HLA_DRB1*01:01:01 + HLA_DRB1*04 + HLA_DRB1*04:01:01:01 + HLA_DRB1*10 + HLA_DRB1*10:01:01:01 + EV1 + EV2 + EV3 + EV4

	t value	Pr(>|t|)
(Intercept)	4.0895502	0.0001099
rs3128921	-5.2004361	0.0000017
HLA_DRB1_01	-1.1721278	0.2449569
HLA_DRB1_01_01_01	1.0904682	0.2790934
HLA_DRB1_04	-0.5288681	0.5985014
HLA_DRB1_04_01_01_01	0.7732922	0.4418463
HLA_DRB1_10	-1.7400171	0.0860703
EV1	-1.6615725	0.1008875
EV2	1.1069942	0.2719309
EV3	-0.4607323	0.6463603
EV4	-0.7340306	0.4652812

DAS28_ESR ~ rs3128921 + HLA_DRB1*01 + HLA_DRB1*01:01:01 + HLA_DRB1*04 + HLA_DRB1*04:01:01:01 + HLA_DRB1*10 + HLA_DRB1*10:01:01:01 + EV1 + EV2 + EV3 + EV4

	t value	Pr(>|t|)
(Intercept)	3.4783875	0.0008981
rs3128921	-3.2473727	0.0018324
Pathotype	2.0072982	0.0488159
HLA_DRB1_01	-1.5474469	0.1265375
HLA_DRB1_01_01_01	1.6433914	0.1050580
HLA_DRB1_04	0.1018905	0.9191525
HLA_DRB1_04_01_01_01	0.7849794	0.4352744
HLA_DRB1_10	0.3321399	0.7408361
EV1	-1.0329506	0.3053973
EV2	0.7026144	0.4847669
EV3	-1.5431527	0.1275753
EV4	-0.3327636	0.7403674

Delta ESR ~ rs3128921 + HLA_DRB1*01 + HLA_DRB1*01:01:01 + HLA_DRB1*04 + HLA_DRB1*04:01:01:01 + HLA_DRB1*10 + HLA_DRB1*10:01:01:01 + EV1 + EV2 + EV3 + EV4		
	t value	Pr(>|t|)
(Intercept)	2.6174678	0.0109735
rs3128921	-3.8895667	0.0002360
Pathotype	1.4394495	0.1547495
HLA_DRB1_01	-0.9192813	0.3612961
HLA_DRB1_01_01_01	1.1346674	0.2606189
HLA_DRB1_04	0.1229120	0.9025503
HLA_DRB1_04_01_01_01	-0.9732915	0.3339622
HLA_DRB1_10	-0.7882452	0.4333754
EV1	-0.3291469	0.7430869
EV2	-0.0130173	0.9896533
EV3	-0.9779493	0.3316705
EV4	0.1218949	0.9033526

VAS ~ rs3128921 + HLA_DRB1*01 + HLA_DRB1*01:01:01 + HLA_DRB1*04 + HLA_DRB1*04:01:01:01 + HLA_DRB1*10 + HLA_DRB1*10:01:01:01 + EV1 + EV2 + EV3 + EV4		
	t value	Pr(>|t|)
(Intercept)	0.9991099	0.3213905
rs3128921	-1.8530080	0.0683524
Pathotype	0.9766793	0.3322943
HLA_DRB1_01	-0.4212747	0.6749233
HLA_DRB1_01_01_01	0.9869131	0.3272896
HLA_DRB1_04	-0.8783627	0.3829335
HLA_DRB1_04_01_01_01	1.0995129	0.2755389
HLA_DRB1_10	0.9241255	0.3587874
EV1	-0.6165617	0.5396448
EV2	0.7568498	0.4518333
EV3	-1.4426410	0.1538508
EV4	0.5934279	0.5549231
